# Self-assembled supramolecular artificial light-harvesting nanosystems: construction, modulation, and applications

**DOI:** 10.1039/d2na00934j

**Published:** 2022-12-31

**Authors:** Xu-Man Chen, Xiao Chen, Xiao-Fang Hou, Shu Zhang, Dongzhong Chen, Quan Li

**Affiliations:** a Institute of Advanced Materials and School of Chemistry and Chemical Engineering, Southeast University Nanjing 211189 China quanli3273@gmail.com; b Key Lab of High Performance Polymer Materials and Technology of Ministry of Education, School of Chemistry and Chemical Engineering, Nanjing University Nanjing 210023 China; c Advanced Materials and Liquid Crystal Institute and Materials Science Graduate Program, Kent State University Kent OH 44242 USA

## Abstract

Artificial light-harvesting systems, an elegant way to capture, transfer and utilize solar energy, have attracted great attention in recent years. As the primary step of natural photosynthesis, the principle of light-harvesting systems has been intensively investigated, which is further employed for artificial construction of such systems. Supramolecular self-assembly is one of the feasible methods for building artificial light-harvesting systems, which also offers an advantageous pathway for improving light-harvesting efficiency. Many artificial light-harvesting systems based on supramolecular self-assembly have been successfully constructed at the nanoscale with extremely high donor/acceptor ratios, energy transfer efficiency and the antenna effect, which manifests that self-assembled supramolecular nanosystems are indeed a viable way for constructing efficient light-harvesting systems. Non-covalent interactions of supramolecular self-assembly provide diverse approaches to improve the efficiency of artificial light-harvesting systems. In this review, we summarize the recent advances in artificial light-harvesting systems based on self-assembled supramolecular nanosystems. The construction, modulation, and applications of self-assembled supramolecular light-harvesting systems are presented, and the corresponding mechanisms, research prospects and challenges are also briefly highlighted and discussed.

## Abstract 2

The recent progress in construction, modulation and applications of nanoscale self-assembled supramolecular light-harvesting systems is briefly reviewed and discussed.

## Graphical abstract

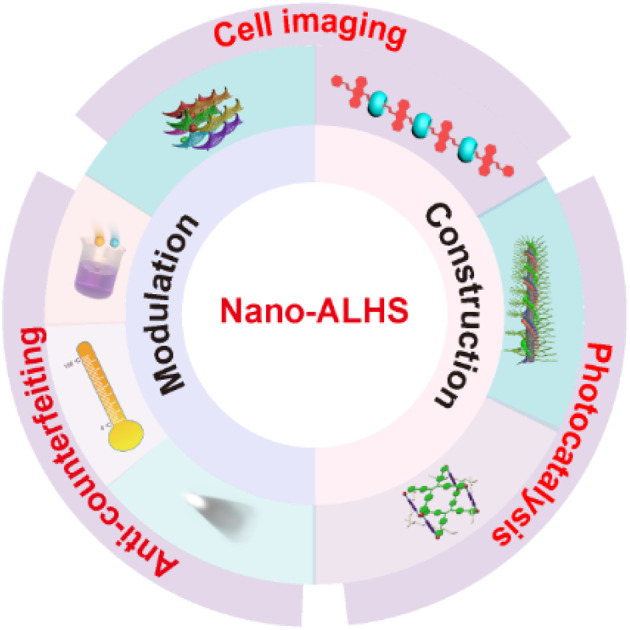

## 1. Introduction

Sunlight is the original source that supplies energy for all the biological activities in the world.^1–4^ A huge amount of sunlight is received by Earth every day; however, only a small part of it is utilized by human beings.^5–12^ Nature always takes advantage of photosynthesis to capture solar energy and construct higher carbon products, which offers great inspiration to fabricate artificial ones according to the principle of photosynthesis. In higher plants and photosynthetic bacteria, light-harvesting antenna supramolecular complexes absorb the solar energy and transfer it through long-range energy funnels to reaction centers, which can further drive the reactions to convert the transferred light energy into chemical energy.^4,13–19^ Nevertheless, from capturing solar energy to finally forming higher carbon products, photosynthesis is a multistep complex process, which has been hard to completely reproduce artificially so far. Thus, at the present stage, mimicking some of the steps of photosynthesis can help us gradually recognize the whole photosynthesis as well as improve the utilization efficiency of solar energy.^20–22^ Among all the steps, light-harvesting is the primary step of photosynthesis, which plays an important role in determining the utilization efficiency of solar energy. Therefore, more and more attention has been focused on developing artificial light-harvesting systems (ALHSs).

Antenna chromophores are employed first to absorb and transfer light energy in common ALHSs. Then, to avoid the dissipation of light energy that is captured by antenna chromophores, Förster resonance energy transfer (FRET) is one feasible process for achieving the energy transfer.^16,23–25^ Thus, the antenna chromophores are referred to as FRET donors, which transfer the absorbed light energy to FRET acceptors to realize the artificial light-harvesting process.^26–32^ According to the mechanism of FRET and the requirements of ALHSs, there are four key considerations: (1) a high donor/acceptor molecular ratio; (2) a large overlap between the emission band of the donor and the absorption band of the acceptor; (3) a distance in the range of 0.1 to 10 nm between donors and acceptors; (4) avoiding emission quenching of donors even though a number of donor chromophores are stacked for energy transfer to one acceptor. Therefore, to construct ALHSs satisfying the above listed points, systems at the nanoscale, *i.e.*, artificial light-harvesting nanosystems are an available and viable choice.

In the past decade, many types of ALHSs have been developed by employing dendrimers,^33–35^ molecular arrays,^36–39^ polymers,^40–47^ anisotropic fluid^48^ and amphiphilic self-assembled structures.^49–57^ Both covalent and non-covalent interactions are used to bind light-harvesting donors and acceptors. Covalent binding between donors and acceptors is a stable linkage for light-harvesting, but the donor/acceptor ratio is hard to reach at the highest level of the natural light-harvesting system (∼200 : 1) because of the too complicated synthesis challenge to covalently link large number of donors to one acceptor.^58,59^ In contrast, non-covalent binding endows ALHSs with more possibilities in a relatively easier way to achieve a high donor/acceptor ratio as well as controllable properties, but a rational design based on strong driving forces is required to achieve high efficiency of FRET.^60,61^ Therefore, different supramolecular construction strategies have been developed to pursue both high donor/acceptor ratios and improved light-harvesting efficiency. Two main factors are chosen to evaluate the light-harvesting efficiency, energy transfer efficiency (*Φ*_ET_) and the antenna effect (AE), which reflect the percentage of energy transferred from the donor and the emission enhancement of the acceptor caused by donor antennas, respectively.^62–64^ In consideration to construct highly efficient ALHSs, supramolecular nanosystems receive extensive attention because donor and acceptor chromophores in such nanoscale supramolecular systems can easily keep a suitable distance in the nanometer range for achieving high transfer efficiency.

Recently, Hu and coworkers have reviewed ALHSs based on macrocycle-assisted supramolecular assembly in aqueous media.^14^ Xiao, Elmes and coworkers have reported ALHSs fabricated *via* supramolecular host–guest interactions.^65^ Moreover, Yang, Xu and coworkers have presented supramolecular ALHSs with a special emission property, aggregation-induced emission (AIE).^60^ Considering recent research progress and previously presented reviews on ALHSs, we think that it is a very important topic to summarize the advances in supramolecular self-assembled light-harvesting nanosystems (nano-ALHSs). In this review, we introduce the recent advances in supramolecular nano-ALHSs from the viewpoints of construction, controllable supramolecular synthesis methods, and applications. Host–guest complexes, biomaterials and metal–organic complexes are common construction structures for nano-ALHSs, which are summarized in detail in the first section. Subsequently, stimuli-responsive and other controlled methods are introduced. Furthermore, some potential applications such as photocatalysis, cell imaging, encryption, and anti-counterfeiting are summarized in the third section. Therefore, in this review article, we aim to provide inspiration for the construction of highly efficient and applicable supramolecular nano-ALHSs. Furthermore, mechanisms, research prospects, and challenges are also briefly discussed.

## 2. Construction of self-assembled supramolecular nano-ALHSs

Construction of supramolecular nano-ALHSs requires a precise design because of the high sensitivity of light-harvesting efficiency at an extremely trace concentration of acceptors, which depends on the complex interactions between the donors, the media, and the acceptors in the ALHSs.^66–79^ In this section, supramolecular nano-ALHSs based on host–guest complexation, biomacromolecules and metal–ligand complexation are summarized because these systems exhibit recognizable, strong, and controllable molecular interactions.

### 2.1 Self-assembled supramolecular nano-ALHSs based on host–guest complexation

**Fig. 1:**
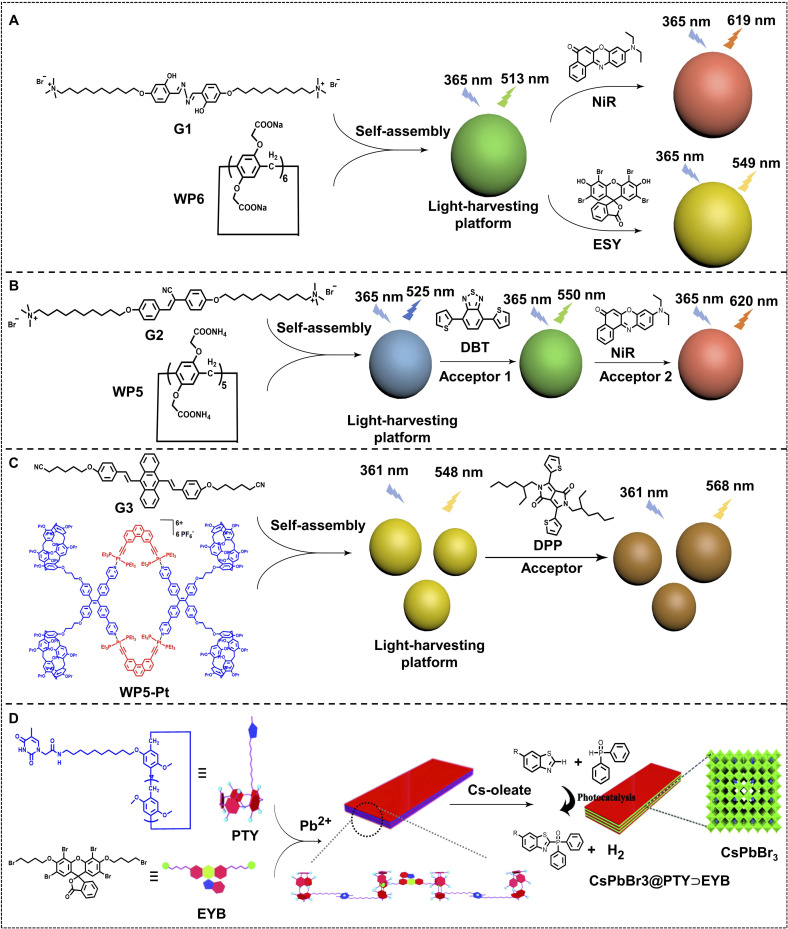
(A) Schematic illustration of the self-assembly of pillar[6]arene-based aqueous nano-ALHSs.^80^ (B) Schematic illustration of supramolecular self-assembly of nano-ALHSs with two-step sequential energy transfer in aqueous solution based on WP5, G2, DBT, and NiR.^81^ (C) Schematic of the structure of rhomboidal metallacycle WP5–Pt, and the chemical structures of building block DSA-containing guest G3 and fluorescent acceptor DPP to form a nano-ALHS. Reproduced with permission from ref. 82. Copyright 2021, Royal Society of Chemistry. (D) Schematic representation of the self-assembly and photocatalytic hydrogen evolution reaction process of a supramolecular CsPbBr_3_@PTY⊃EYB nano-ALHS. Reproduced with permission from ref. 83. Copyright 2021, Royal Society of Chemistry.

Host–guest complexation *via* pillararenes is an effective pathway for constructing nano-ALHSs. As shown in Fig. 1A, Wang, Hu and coworkers synthesized a salicylaldehyde azine derivative (G1) as a light-harvesting donor, which can co-assemble with a pillar[6]arene carboxylate (WP6) to construct an artificial light-harvesting platform.^80^ Such a light-harvesting platform exhibited spherical nanoparticles (average diameter: 109.2 nm) and enhanced the emission of the donor G1 by 28 times. The G1–WP6 co-assembly is a versatile artificial light-harvesting platform that can transfer its emission energy to two different acceptor fluorophores, Nile red (NiR) and Eosin Y (ESY). When loading trace amounts of NiR as well as ESY into G1–WP6 nanoparticles, both the two ALHSs work at high efficiency. The AEs of the two ALHSs are calculated to be 25.4 for the NiR loaded ALHS at a 150 : 1 donor/acceptor ratio and 28.0 for the ESY loaded ALHS at a 200 : 1 donor/acceptor ratio.

Furthermore, they developed a highly efficient nano-ALHS based on a two-step sequential FRET supramolecular self-assembly system based on self-assembly between a bola-type bis(4-phenyl)acrylonitrile derivative (G2) and pillar[5]arene carboxylate (WP5). (4,7-Bis(thien-2-yl))-2,1,3-benzothiadiazole (DBT) and NiR were selected as two sequential FRET acceptors which can be loaded in G2–WP5 self-assemblies to realize an efficient ALHS (Fig. 1B).^81^ In this ALHS system, the emission energy of G2 effectively transfers to DBT at a 350 : 1 molar ratio, which is referred to as a light-harvesting process, while the second stage of FRET is realized at [DBT] : [NiR] = 1 : 1 at the same time. The two-stage G2–WP5–DBT–NiR light-harvesting system exhibited pure white-light emission at a certain concentration ([WP5] = 2 × 10^−5^ M, [G2] = 1 × 10^−4^ M, [DBT] = 2.8 × 10^−7^ M, and [NiR] = 2.1 × 10^−7^ M, respectively), which was capable of white fluorescence emission and promising to be applied for visible-light photocatalysis as well as mimicking the multi-step energy transfer process in nature.

Beyond nano-ALHSs based on the host–guest complexation between anionic pillararene and cationic ammonium in aqueous solution, the molecular recognition between pillararene and alkyl nitriles in non-aqueous solution can also be used for constructing nano-ALHSs.^82^ As shown in Fig. 1C, a rhomboidal metallacycle bearing four pillar[5]arene macrocycles WP5–Pt was synthesized as a host building block to construct a light-harvesting platform with 9,10-distyrylanthracene-containing dinitrile guest G3 *via* host–guest complexation in THF/water. A hydrophobic fluorophore diketopyrrolopyrrole DPP was selected as a light-harvesting acceptor to construct a (WP5–Pt⊃G3)⊃DPP dual-donor nano-ALHS. Such a nano-ALHS has taken advantage of metal–ligand coordination, host–guest complexation and hydrophobic interactions, and it exhibits an excellent capacity for harvesting both UV and blue light. The *Φ*_ET_ and AE reached 53.1% and 12.8, respectively, which indicates that such a strategy for constructing nano-ALHSs has great potential for applications.

The host–guest complexation between pillararenes and alkyl chains has been also utilized for constructing metal–organic nano-ALHSs.^83^ As shown in Fig. 1D, CsPbBr_3_ quantum dots were synthesized as light-harvesting donors, whose emission band showed a good overlap with that of a common fluorescent acceptor, ESY. Thus, a two bromo-butyl modified ESY derivative (EYB) was synthesized as a guest fluorophore to bind with a thymine functionalized-pillar[5]arene (PTY) for preparing a supramolecular polymer. Such an EYB–PTY based supramolecular polymer further assembled with CsPbBr_3_ quantum dots to form an efficient light-harvesting system. The obtained light-harvesting system showed a high *Φ*_ET_ of up to 96.5% and displayed excellent photocatalytic activity in cross-coupling hydrogen evolution reactions, which led to more than 2.5 times the product yield than using only EYB itself for the same catalytic reaction. Therefore, such a two-stage self-assembled nano-ALHS based on host–guest complexation and metal–organic complexation provides a new pathway for photocatalysis with solar energy.

**Fig. 2:**
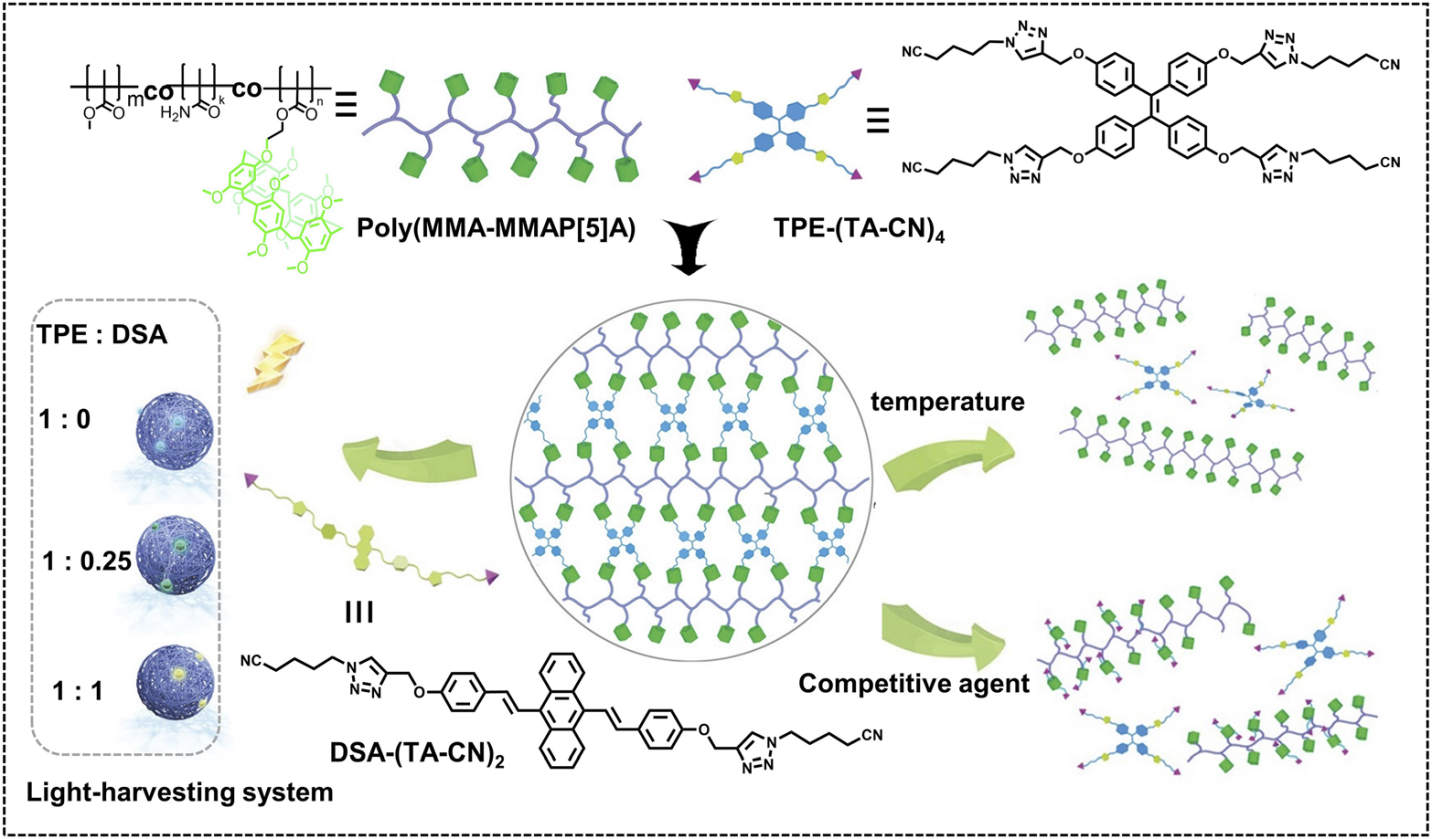
Schematic illustration of the construction and property study of supramolecular polymer networks (SPNs) based on the copolymer poly(MMA–MMAP[5]A) and two AIEgens (TPE and/or DSA), which could be further assembled into supramolecular nanoparticles for the fabrication of light-harvesting systems. Reproduced with permission from ref. 43. Copyright 2019, Wiley-VCH.

Pillararenes can be further tethered to synthetic polymers as a medium to construct nano-ALHSs. Yang, Tang and coworkers reported a nano-ALHS based on a newly synthesized linear copolymer with a number of pillar[5]arene dangling side chains acting as hosts (Fig. 2).^43^ Based on the host–guest interactions between pillar[5]arene and cyano groups and the AIE behavior of tetraphenylethene (TPE), the authors synthesized a TPE-based guest molecule and complexed such a guest molecule with pillararene modified polymers to form spherical supramolecular nanoparticles through host–guest complexation. The thus obtained supramolecular nanoparticles exhibited an extremely high fluorescence quantum yield of 98.22% in THF. Then, another luminophore, 9,10-distyrylanthracene (DSA), was introduced into the supramolecular nanoparticles as an acceptor to achieve an efficient nano-ALHS. The quantum yield of the ALHS was measured to be 51.69% after introducing a preferable amount of the acceptor. As the molar ratio between the donor and acceptor reached 1 : 0.25, the *Φ*_ET_ reached as high as 88.34% and the AE value was calculated to be 3.63. Furthermore, *Φ*_ET_, AE, emission and quantum yields of such ALHSs can all be tuned by controlling the solvent as well as the donor/acceptor ratio.

**Fig. 3:**
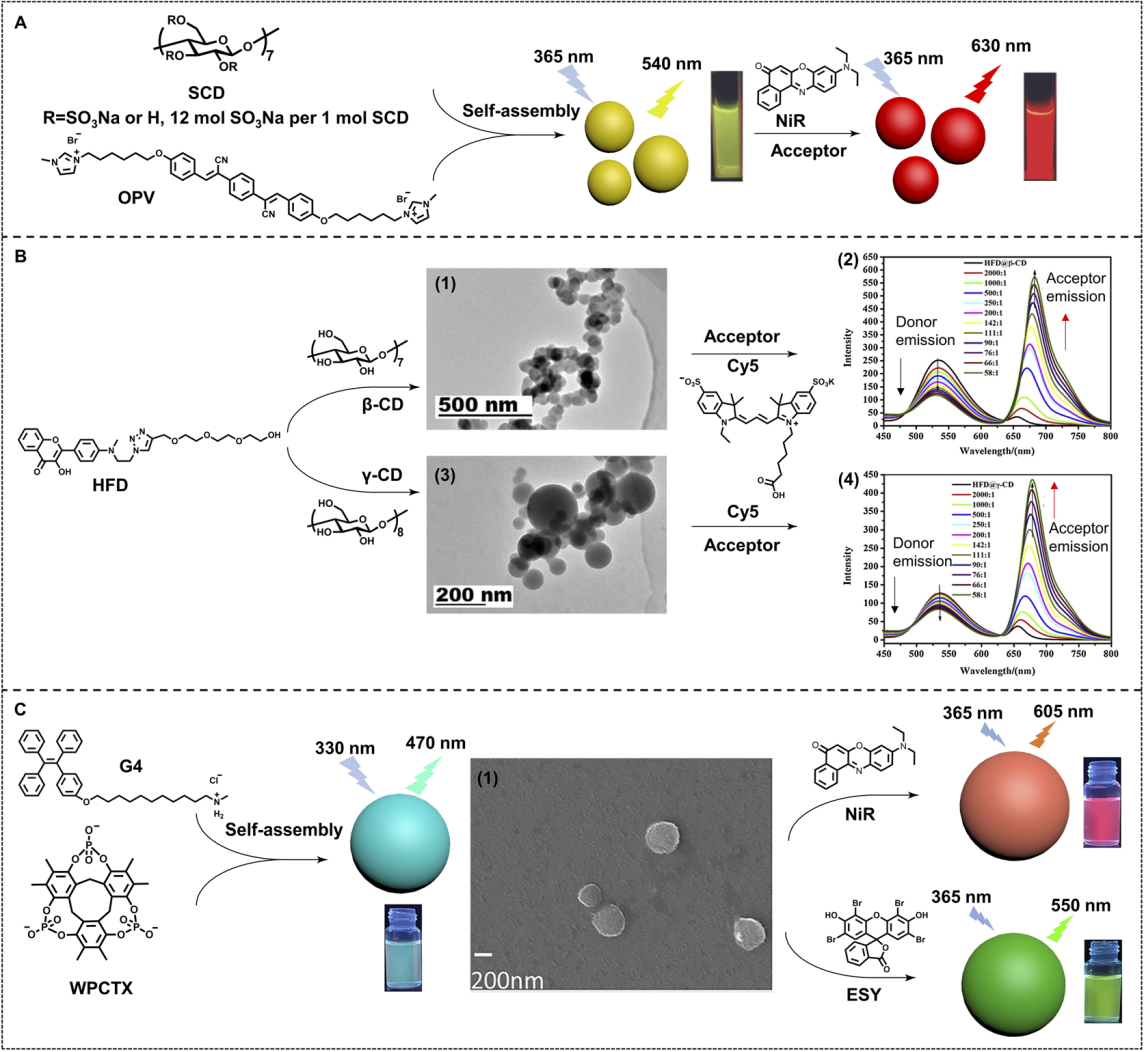
(A) Schematic illustration of a highly efficient OPV/SCD/NiR supramolecular nano-ALHS. Reproduced with permission from ref. 55. Copyright 2017 Wiley-VCH. (B) Chemical structures of the building blocks of aqueous nano-ALHSs, and TEM images of assemblies formed by (1) HFD@β-CD and (3) HFD@γ-CD. Fluorescent emission spectra of (2) HFD@β-CD and (4) HFD@γ-CD with an increasing concentration of Cy5. Adapted with permission from ref. 84. Copyright 2021, Elsevier. (C) Chemical structures of G4 and WPCTX, (1) SEM image of assemblies formed by G4 and WPCTX, and the variation in fluorescent color on construction of nano-ALHSs using NiR and ESY as acceptors respectively. Adapted with permission from ref. 85. Copyright 2021, Wiley-VCH.

Cyclodextrin is also a promising macrocycle for fabricating efficient nano-ALHSs. Liu and coworkers constructed an efficient nano-ALHS by utilizing a cyclic polysaccharide, sulfonato-β-cyclodextrin (SCD), an oligo(phenylenevinylene) derivative (OPV) and NiR. OPV was employed as the donor of the ALHS and transferred the light energy to NiR (Fig. 3A).^55^ OPV and SCD co-assembled into ∼100 nm spherical nanoparticles through electrostatic and amphiphilic interactions. The constructed supramolecular nanoparticles enhanced the fluorescence of OPV by several times as well as provide loading sites for NiR, ensuring an efficient FRET process between the two fluorophores. The FRET process started to happen at an extremely high donor/acceptor molar ratio (125 : 1), which was considered an efficient ALHS system. Such an OPV–SCD–NiR system showed both a high *Φ*_ET_ (72%) and AE (32.5).

The host–guest complexation based on cyclodextrin for further self-assembly is also an effective pathway for fabricating nano-ALHSs. As shown in Fig. 3B, an amphiphilic hydroxyflavone derivative (HFD) with a hydrophilic side chain modification was synthesized as the light-harvesting donor, which can co-assemble with both β- and γ-cyclodextrin (β-CD and γ-CD) to form two different light-harvesting platforms.^84^ After this, a typical fluorophore, Cy5, was employed as the light-harvesting acceptor to be loaded into the two types of donor assemblies. Although α-cyclodextrin (α-CD) cannot include the HFD because its cavity volume is too small for a HFD molecule, the HFD can still co-assemble with α-CD and the HFD@α-CD co-assembly can also be constructed into HFD@α-CD–Cy5 ALHSs. The three kinds of ALHSs (HFD@α-CD–Cy5, HFD@β-CD–Cy5, and HFD@γ-CD–Cy5) showed both high *Φ*_ET_ (34.8%, 54.6% and 24.5%) and AE (15.1, 16.6 and 12.8), respectively, which provided a promising design for highly efficient nano-ALHSs through controlling fluorescence intensity and changing the size of self-assemblies.

Moreover, as a kind of brand-new synthetic macrocycle, a water-soluble phosphate-based cyclotrixylohydroquinoylene (WPCTX) was reported for investigation of light-harvesting ability.^85^ As shown in Fig. 3C, WPCTX was successfully synthesized through three-step reactions. The new anionic macrocycle exhibits obvious host–guest complexation ability for ammonium as well as pyridinium groups. The association constant was calculated to be up to (284 ± 11) M^−1^, which indicated the rather good capacity of host–guest complexation between WPCTX and these cationic groups in aqueous solution. Thus, an amphiphilic tetraphenylethene ammonium derivative (G4) has been synthesized to assemble with WPCTX to form AIE co-assemblies through host–guest complexation and amphiphilic interactions. Such co-assemblies can act as a light-harvesting platform, which exhibits highly efficient energy transfer behavior to both ESY and NiR, two typical light-harvesting acceptors, at a high donor/acceptor ratio (150 : 1 for ESY and 100 : 1 for NiR). Both high *Φ*_ET_ (50.1% and 58.9%) and AE (9.1 and 11.0) are obtained from G4–WPCTX–ESY and G4–WPCTX–NiR light-harvesting systems, respectively.

### 2.2 Self-assembled supramolecular nano-ALHSs based on biomacromolecules

With regard to construction of nano-ALHSs, the first step is to build up the supramolecular nanoparticles as a platform based on non-covalent interactions for the energy transfer process to take place. Applying non-toxic biomacromolecules directly to assemble into supramolecular nanoparticles is a fascinating method, and makes the variation of luminescence more important for the great potential to be applied in living cells, whether in the fields of biosensing, cell imaging, drug delivery, gene delivery, *etc.*

Recently, researchers all around the world have constructed various supramolecular assemblies based on DNA, proteins, peptides and some other biomacromolecule such as synthesized polymers.

Light-harvesting complexes capture light energy and deliver it, and finally transform it into chemical energy. Construction of light-harvesting systems with DNA and various luminophores (different luminophores acting as donors and acceptors respectively) has the following advantages: (1) DNA is a natural biomacromolecule with high biocompatibility; (2) the double stranded structure of DNA as a supramolecular scaffold can separate the luminophores and reduce the possibilities of the quenching behavior, making it easier to achieve high energy transfer efficiency and antenna effect; (3) the non-covalent interactions between DNA enables the supramolecular assemblies with stimuli responsive properties, making the supramolecular light-harvesting system more precisely controlled by different stimuli; (4) the helical structure and DNA-templated assembly behavior sometimes can induce the chirality of assemblies, together with variation in circularly polarized luminescent signals.

**Fig. 4:**
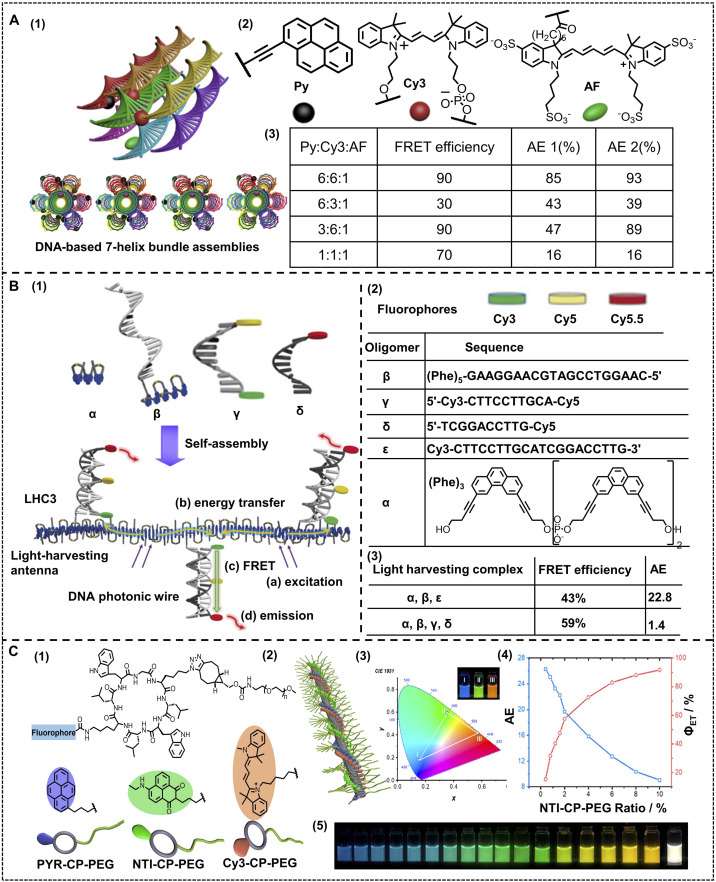
(A) (1) Schematic representation of the four triads. (2) Chemical structure of the light-harvesting chromophores. (3) *Φ*_ET_ and AE of the light-harvesting systems with different donor/acceptor molar ratios. Reproduced with permission from ref. 86. Copyright 2011, American Chemical Society. (B) (1) Schematic illustration of the supramolecular assembly of a light-harvesting complex LHC3. Chromophore-modified DNA hybrids containing terminal phenanthrenes (shown in blue) are incorporated into the growing phenanthrene antenna during supramolecular polymerization. (2) Oligomers used in this study and the chemical structure of phosphodiester-linked phenanthrenes. Bottom: oligomer composition of the different ALHSs. (3) Average FRET efficiency (*Φ*_ET_) and the overall antenna effect (AE) of different ALHSs. Reproduced with permission from ref. 87. Copyright 2019, Wiley-VCH. (C) (1) Artificial light-harvesting system based on supramolecular peptide nanotubes in water: chemical structures of three fluorophore–cyclic peptide–polymer conjugates. (2) Cartoon illustration of the artificial light-harvesting system. (3) CIE 1931 diagram showing CIE coordinates of I, II, and III. White triangle: the color area could be obtained using the three-component system. Inset: photograph of I, II, and III. (4) AE and *Φ*_ET_ at different NTI–CP–PEG/PYR–CP–PEG ratios. (5) Photograph showing different emission colors at different PYR–CP–PEG/NTI–CP–PEG/Cy3–CP–PEG ratios. Reproduced with permission from ref. 88. Copyright 2021, American Chemical Society.

Liu and coworkers employed DNA as the assembly scaffold, where three distinct chromophores were introduced into a nano-ALHS, with ethynylpyrene (Py) acting as a primary donor array and a cyanine-derived dye (Cy3) acting as a secondary or intermediate donor, together with an Alexa Fluor 647 (AF) acting as the acceptor (Fig. 4A).^86^ Benefiting from the well-defined DNA-templated assembly and the three distinct chromophore arrays, seven triad configurations with precise interchromophore distance and a well-defined donor–acceptor ratio were established. As the ratio among Py : Cy3 : AF in the ALHS was 6 : 6 : 1, energy transfer efficiency reached 90%, which is a significant high light-harvesting efficiency. This work successfully demonstrated that DNA is an excellent platform to organize arrays of distinct chromophores to further construct high-efficiency light-harvesting supramolecular assemblies with stimuli responsive properties. Such a feasible control was realized only with a convenient variation in the ratio between distinct dyes, which could greatly modulate the light-harvesting efficiency. Different from the way of simply complexing luminophores with DNA to construct artificial light-harvesting systems, Häner and coworkers reported a series of light-harvesting supramolecular phenanthrene-containing polymer hybrids with DNA as a scaffold to assemble and various luminophores are covalently linked to a series of DNA acting as photonic wires (Fig. 4B).^87^ In this work, four different fluorophores, phenanthrene, Cy3, Cy5 and Cy5.5 are employed. A phenanthrene containing oligomer acts as a primary donor, while a series of different dye labelled oligonucleotides, that is covalently substituted DNA scaffolds, acted both as assembly blocks and block containing acceptors. The authors complexed different dye-labeled DNA with the primary donor and successfully constructed a high efficiency light-harvesting system. Owing to the amphiphilic properties of DNA, the non-covalent interactions and the backbone provided by DNA chains, the complex successfully assembled with these separated dyes, which restrained the aggregation and caused quenching to achieve remarkable energy transfer efficiency. Such an ALHS from a simple complexation of a phenanthrene oligomer and Cy3-labeled DNA achieved an AE of 22.8. Among the obtained complexes, a significant light-harvesting assembly formed by the phenanthrene oligomer, Cy3-labeled DNA, Cy5-labeled DNA and Cy5.5-labeled DNA achieved an *Φ*_ET_ as high as 59%. The high *Φ*_ET_ was not only relevant to the well-organized state of assembly benefiting from DNAs, but also owing to the stepwise energy transfer process from the primary energy donor, phenanthrene, to the intermediate acceptors, Cy3 and Cy5, then finally to a Cy5.5 acceptor through a FRET mechanism.

**Fig. 5:**
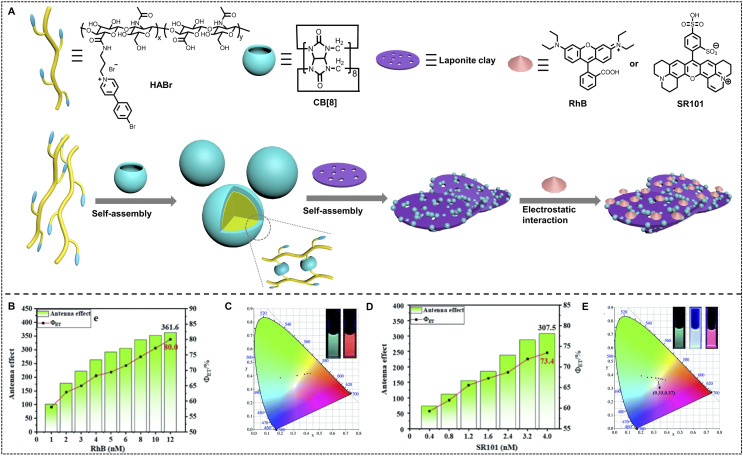
(A) Schematic illustration of the construction of RTP light harvesting by the multivalent supramolecular assembly HABr/CB[8]/LP with RhB or SR101 in aqueous solution. (B) The antenna effect/FET of HABr/CB[8]/LP in aqueous solution with different concentrations of RhB (according to the emission of the donor: 500 nm and acceptor 1: 585 nm). (C) The CIE chromaticity diagrams of the photoluminescence color changes from varying the ratios of RhB (inset: photographs of HABr/CB[8]/LP and HABr/CB[8]/LP/RhB). (D) The antenna effect/FET of HABr/CB[8]/LP in aqueous solution with different concentrations of SR101 (according to the emission of the donor: 500 nm and acceptor 2: 612 nm). (E) The CIE chromaticity diagrams of the photoluminescence color changes by varying the ratios of SR101 (inset: photographs of HABr/CB[8]/LP, HABr/CB[8]/LP/0.005SR101 and HABr/CB [8]/LP/SR101). Reproduced with permission from ref. 89. Copyright 2021, Royal Society of Chemistry.

The related studies demonstrated that DNA is an excellent building block to fabricate nano-ALHSs and has great potential to be further used in various applications due to its stimuli-responsive properties. Besides DNA, other biomacromolecules such as proteins and peptides have also been developed in supramolecular nano-ALHSs. Perrier and coworkers reported an ALHS mediated by a cyclic peptide polymer (Fig. 4C).^88^ Based on a careful investigation of the absorption and emission spectra of fluorophores, the authors selected three types of dyes, and then synthesized three assembly blocks, pyrene–cyclic peptide–poly(ethylene glycol), cyanine3–cyclic peptide–poly(ethylene glycol) and naphthalene monoamide–cyclic peptide–poly(ethylene glycol), by covalently modifying the dyes to the peptides. Due to the relative strong interactions between cyclic peptides, especially the existence of strong hydrogen bonding between the flat rings of the peptides and the exceptional amphiphilic properties that peptides exhibit, the dye-modified cyclic peptides formed stable co-assemblies in aqueous media. Moreover, owing to the covalent connection of the dyes to the peptides, the stacking of fluorophores was greatly reduced; thus significantly avoiding the aggregation induced quenching effect. Such stable co-assemblies in aqueous solution maintained an appropriate distance between the three dyes for the two-step FRET process and realized high efficiency of the ALHS. By simply adjusting the molar ratio between the three types of dye-modified peptides carefully, the light-harvesting system achieved a quantum yield of over 30% in aqueous media. Interestingly, the emission color of such an ALHS was successfully tuned continuously from blue to green and finally orange including pure white emission with a quantum yield of up to 29.9%. Remarkably, such an ALHS can work stably whether at an extremely low concentration (<1 μM) or at relatively high temperature (>80 °C). This work revealed that peptides with strong interactions and amphiphilic characters are exceptional assembly blocks to construct a highly emissive light-harvesting system and mimic the photosynthesis process in nature. Amphiphilic polysaccharides can also be employed to construct artificial light-harvesting systems beyond DNA and peptides. Liu and coworkers reported a supramolecular nano-ALHS by complexation of the following components: 4-(4-bromophenyl)pyridine-1-ium bromide modified hyaluronic acid, cucurbit[8]uril and LAPONITE® clay (Fig. 5).^89^ The successfully constructed multivalent supramolecular assemblies exhibited phosphorescence emissive properties enabling the assemblies to act as donors themselves, while two distinct dyes, rhodamine B (RhB) or sulfonated rhodamine 101 (SR101) were loaded into the luminescent assemblies as acceptors. Due to the existence of 4-(4-bromophenyl)pyridine-1-ium bromide, the supramolecular assemblies could exhibit wholly organic room-temperature phosphorescence with a phosphorescence lifetime of up to 4.79 ms in aqueous solution. It was revealed that there existed a large band overlap between the spectra of the emission of the co-assemblies and the absorption of RhB and SR101. Thus, the luminescent supramolecular assembly showed universal potential as an energy donor. Upon loading RhB into the assembly as an acceptor, when the ratio between the donor and acceptor reached 25 : 1, the *Φ*_ET_ of the system remarkably reached 80% and the AE value reached 361.6. When SR101 was loaded into the system and the ratio between the donor and acceptor reached 75 : 1, the *Φ*_ET_ reached 73.4% and the AE value reached 307.5. Furthermore, by adjusting the molar ratio between the donor and acceptor, a wide range of emission color from blue to orange was achieved when loading RhB, and the emission color was changed from blue, to white, and all the way finally to pink when SR101 acted as the acceptor. Finally, the multicolor emissive nano-ALHS was applied as smart luminescent inks for potential encryption materials.

### 2.3 Self-assembled supramolecular nano-ALHSs based on metal–ligand interactions

To fabricate a supramolecular nano-ALHS, the first step is to construct a platform, for example, supramolecular assemblies at the nanoscale, for the FRET process to take place. As for the driving force to form supramolecular assemblies, apart from host–guest interactions mediated by various macrocycles and the non-covalent interactions among intra/inter-macromolecules, coordination interactions between various metal ions and ligands are also important and commonly used. Coordination-induced self-assembly is an important strategy in the construction of supramolecular assemblies, not only because of their strong, directional, stable and specific properties, but also for the usually well-defined shapes and nanoscale sizes of the generated assemblies. More importantly, with the metal ions involved in the self-assembly system it enables such assemblies to have the capabilities of magnetic, optic, and electronic properties, and catalytic potential. Furthermore, the introduction of the coordination interactions endows the supramolecular system with stimuli-responsive properties. The solvent, ligands, or introduction of other metals can all act as variables to tune the properties of the nanosystem. The rigid, orderly packing of ligands and metals, together with complexed luminophores in the system makes the fabrication of efficient ALHSs extremely easy.

**Fig. 6:**
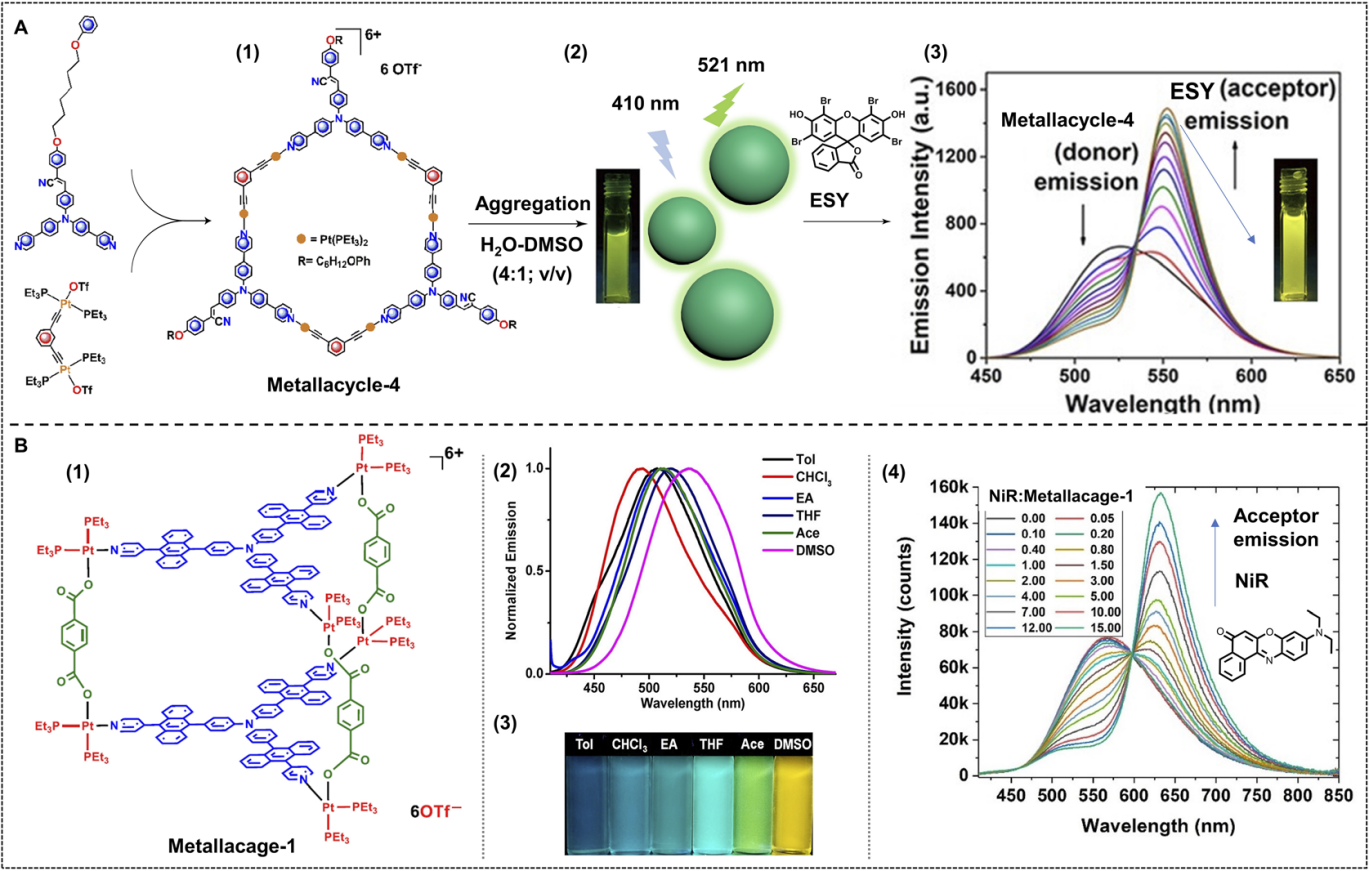
(A) (1) Chemical structure of metallacycle-4 and the relevant precursors. (2) Illustration of nanoparticles constructed by metallacycle-4 in H_2_O–DMSO (4 : 1; v/v), and the emission colour of assemblies in solution under 365 nm UV light excitation. (3) Change in fluorescent emission spectra as the concentration of acceptor, ESY increases. Reproduced with permission from ref. 66. Copyright 2021, American Chemical Society. (B) (1) Chemical structure of metallacage-1. (2) Normalized fluorescent emission spectra of metallacage-1 in different solvents under 395 nm excitation. (3) Photographs of metallacage-1 in the corresponding solvents under 365 nm light. (4) Fluorescent emission spectra variation as the ratio between metallacage-1 and NiR decreases under 400 nm excitation. Reproduced with permission from ref. 90. Copyright 2021, American Chemical Society.

Stang, Acharyya and coworkers reported fluorescent hexagonal Pt(ii) metallacycles with planar structures as a new platform and fabricated a nano-ALHS by combination with a typical light-harvesting acceptor, ESY.^66^ The metallacycles were constructed from a triphenylamine-based ditopic ligand and two di-platinum metallic centers as illustrated in Fig. 6A. The fabricated hexagonal metallacycles possess strong fluorescent emission properties in both solution and solid states. Remarkably, the luminescent metallacycles showed strong AIE properties in a mixture of DMSO and water, and could well self-assemble into spherical nanoparticles in H_2_O–DMSO (4 : 1; v/v) with an average diameter of ∼250 nm, as validated by TEM and SEM investigations. These properties made the metallacycles serve as an exceptional light-harvesting platform and at the same time as the donor. Considering the large overlap between the emission spectra of metallacycles and the absorption spectra of luminophore dyes, a commercial fluorophore ESY was applied as the acceptor. When the molar ratio between the donor and acceptor was 10 : 1 in H_2_O–DMSO (4 : 1; v/v), the energy transfer efficiency reached as high as 65%.

Apart from metallacycles that are driven by the coordination interactions between metal centers and ligands, metallacages, three-dimensional coordination structures, are newly synthesized and further applied in the construction of nano-ALHSs (Fig. 6B) as reported by Stang, Deria and Li *et al.*^90^ In this work, the authors have designed and synthesized two Pt(ii) metallacages based on triphenylamine and anthracene, the assembly of which was driven by coordination interactions. The two Pt(ii) metallacages were constructed by two anthracene-triphenylamine-based tripyridyl ligands as two faces, three dicarboxylates with different length rigid spacers as pillars and six 90° Pt(ii) acceptors as linkers. The fluorescent emission properties of metallacages can be easily tuned by polarity of solvents, temperature, and concentration. The emission color of metallacages can be continuously tuned from deep blue (in toluene) to bright yellow (in DMSO) under a UV lamp. Considering that the emission maxima of the metallacage is right at 554 nm, which is exactly close to the absorption of a commonly used commercial dye, NiR, NiR was chosen as the acceptor for further constructing the light-harvesting system. When NiR was loaded into the assemblies of the metallacages in DMSO, the energy transfer efficiency reached as high as 93% from the donor to the acceptor. This work has inspired researchers to consider a new aspect to construct highly efficient ALHSs and to find a way to offer a rigid scaffold for the energy transfer process to take place.

## 3. Modulation of self-assembled supramolecular nano-ALHSs

The development direction of materials science in recent years has been to find smart materials or intelligent materials, which can change their behavior by responding to external stimuli such as light, pH, temperature, force, electric fields, *etc.*^3,91–94^ These smart materials have played an important role in many fields such as biosensing, automobiles, building and construction, the aviation industry, and electronic and medical applications.^95–100^ Therefore, the modulation of light-harvesting systems obtains researchers’ intensive attention and will be the next popular topic in this field.

### 3.1 Light-controlled supramolecular nano-ALHSs

**Fig. 7:**
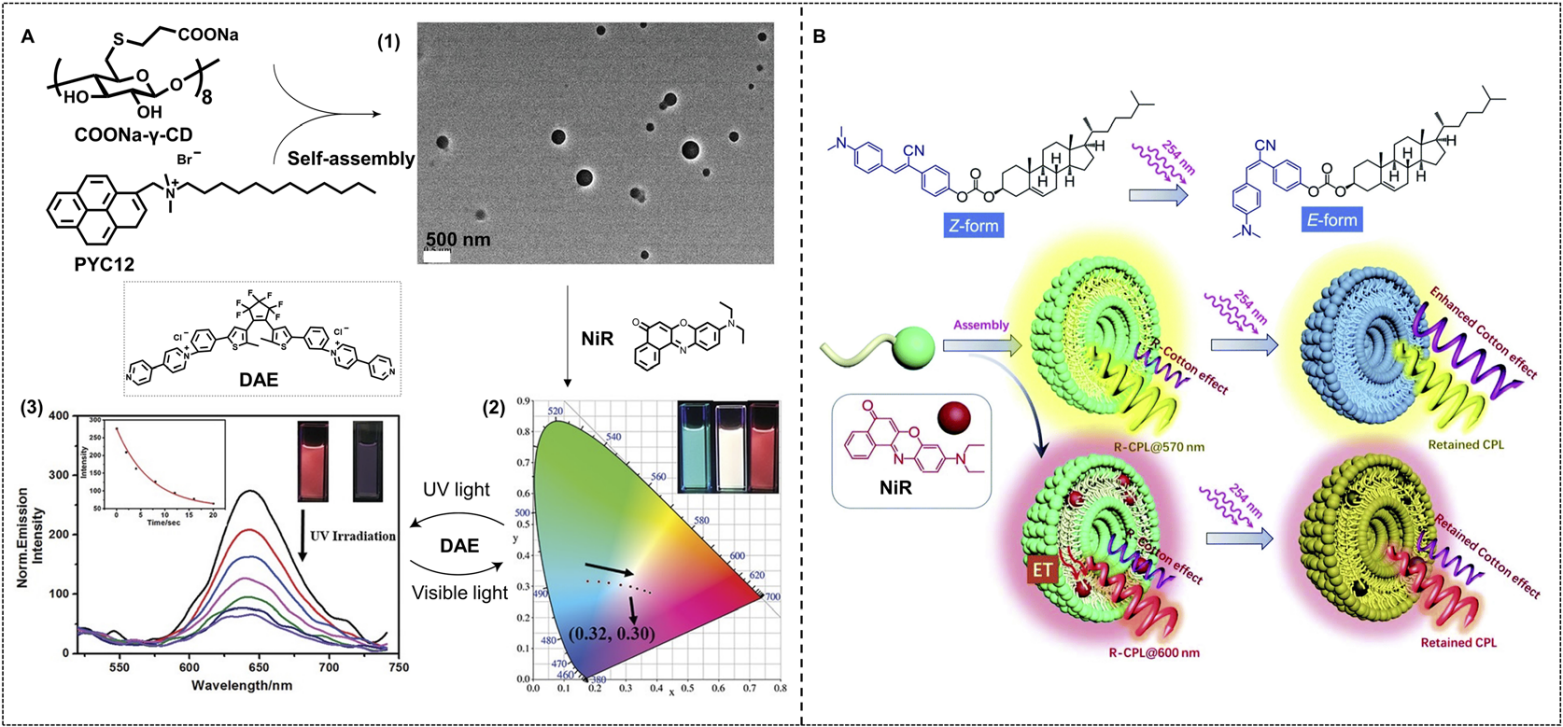
(A) Fabrication of a nano-ALHS through luminescent assemblies formed by PYC12, COONa-γ-CD, and the sequential energy transfer process using NiR and DAE as acceptors. (1) TEM image of nano-assemblies formed by PYC12 and COONa-γ-CD. (2) Variation in the CIE chromaticity diagram as the concentration of NiR increases in the aqueous solution including assemblies. (3) Fluorescent emission spectra of PYC12@COONa-γ-CD@NiR@DAE upon irradiation with 254 nm light. Reproduced with permission from ref. 101. Copyright 2021, Wiley-VCH. (B) Schematic representation of the molecular structure of CSC and NiR as well as their assembling into chiroptical vesicles with light responsiveness. Reproduced with permission from ref. 102. Copyright 2021, Royal Society of Chemistry.

As for self-assembled supramolecular nanosystems, light is a simple but efficient tool to achieve regulation of light-harvesting efficiency. Liu and coworkers have reported a light-controlled supramolecular nano-ALHS constructed with four building blocks including polyanionic γ-cyclodextrin (COONa-γ-CD), a pyrene derivative (PYC12), NiR, and a diarylethene (DAE) derivative in aqueous solution (Fig. 7A).^101^ PYC12 exhibited fluorescence emission at 375 nm and 395 nm, while its emission in the excimer state was red-shifted to around 490 nm after co-assembling with COONa-γ-CD. Thus, PYC12 has become a remarkable energy donor in the PYC12/COONa-γ-CD co-assembled light-harvesting platform. Subsequently, NiR was selected as an energy acceptor and loaded into the PYC12/COONa-γ-CD supramolecular assembly. A highly efficient energy transfer process occurred in this system, and the optimal donor/acceptor ratio is 160 : 1 with an energy transfer efficiency of up to 83%. Benefiting from this ALHS, multicolor containing pure white light-emitting hydrogels were successfully achieved by tuning the molar ratios of the donor and acceptor. With the addition of NiR, the luminescent color changed from cyan to red, and white-light emission with the CIE 1931 chromaticity coordinates of (0.32, 0.30) was also achieved. In order to achieve the photoswitchable fluorescence behavior of this system, a secondary acceptor DAE was loaded into the PYC12/COONa-γ-CD/NiR light harvesting system. DAE possesses an excellent photoisomerization ability between open and closed forms, which can endow the DAE-loaded PYC12/COONa-γ-CD/NiR supramolecular light-harvesting system with controllability by UV light and >600 nm visible light. Under UV light irradiation, the emission of NiR at 640 nm decreased and then recovered upon >600 nm visible light irradiation due to the energy transfer between CF-DAE and PYC12/COONa-γ-CD/NiR.

Circularly polarized luminescence (CPL), one important feature of chiral molecular systems, has been used widely in display, sensing, and information storage. Tunable CPL systems have been well developed *via* supramolecular assembly strategies, and supramolecular nano-ALHSs provided a special pathway for tunable CPL systems (Fig. 7B).^102^ Xing, Hao and coworkers reported a photoresponsive supramolecular nano-ALHS matrix with tunable chiroptical properties. Cholesterol-appended cyanostilbene (CSC) was selected as a luminophore with a chiral center, which can transform from *Z*- into *E*- form under UV light irradiation. The CSCs self-assembled into supramolecular vesicles with exciton Cotton effects and CPL which exhibited a high dissymmetry *g*-factor at a 10^−2^ order of magnitude. After UV light irradiation, Cotton effects of CSC further enhanced by about 3-fold, while the CPL dissymmetry factor was almost unchanged after light irradiation. Subsequently, NiR was used as an acceptor to allow for energy and chirality transfer with luminescence as well as CPL color change from orange to red while retaining a high *g*_lum_ value. Furthermore, the photo-responsiveness of the NiR loaded CSC ALHS was investigated. Under 380 nm UV irradiation, Cotton effects show a merely slight variation, but fluorescence and CPL exhibit a remarkable decrease when 5% NiR was loaded in CSC supramolecular assemblies, which provides a new direction for designing smart chiroptical materials in water.

### 3.2 Thermo-responsive supramolecular nano-ALHSs

**Fig. 8:**
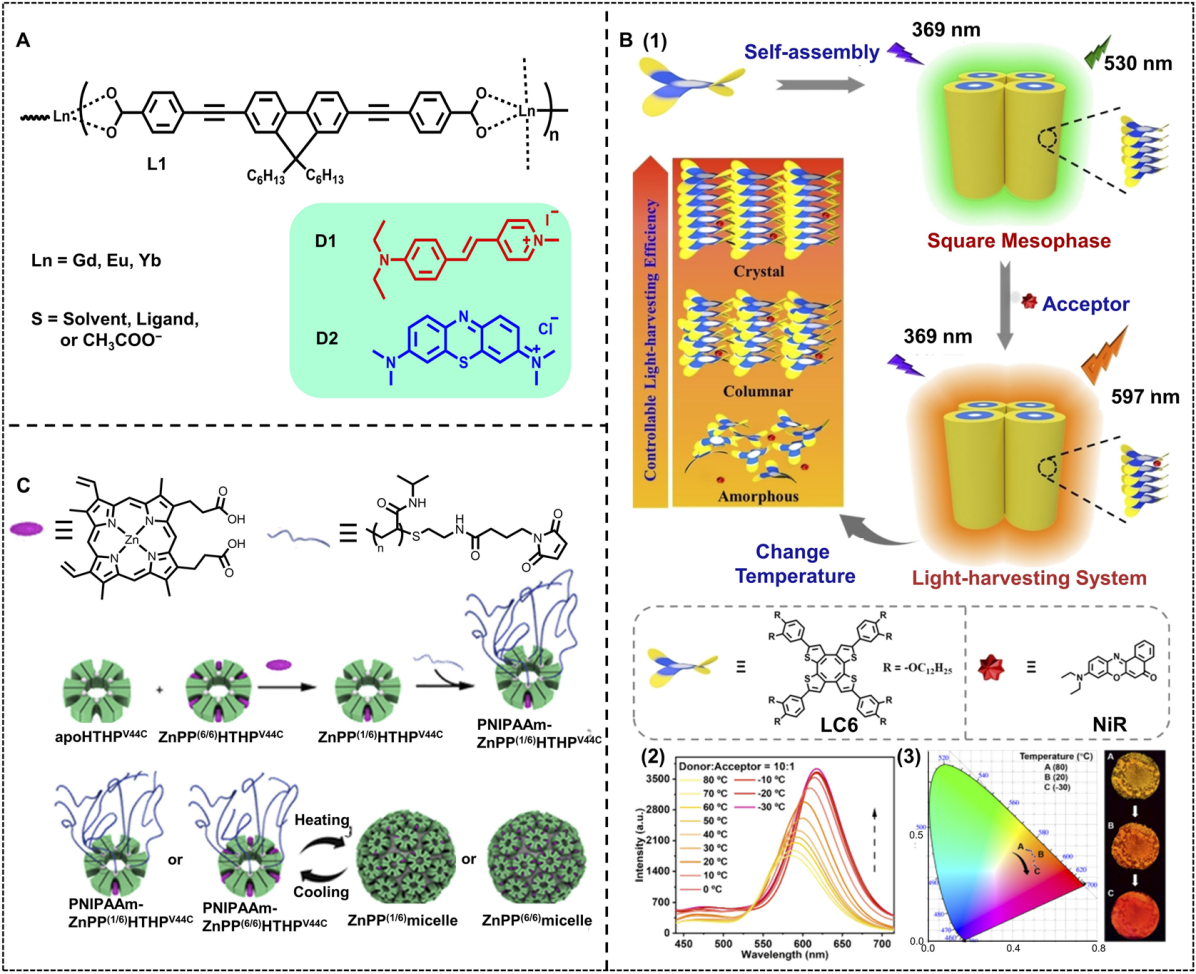
(A) Molecular structures of ligand L1 and guests encapsulated in NCPs. Adapted with permission from ref. 106. Copyright 2010, American Chemical Society. (B) (1) Schematic illustration of the artificial light-harvesting system based on columnar LC6. (2) Fluorescence spectra of LC6-NiR at a donor/acceptor molar ratio of 10 : 1 in a condensed state, *λ*_ex_ = 369 nm. (3) The 1931 CIE chromaticity diagram, which shows the luminescence color coordinates changes of LC6-NiR at different temperatures of (2). Inset: the corresponding fluorescence images at 80 °C (top), 20 °C (middle), and −30 °C (bottom) under 365 nm light. Reproduced with permission from ref. 48. Copyright 2022, Wiley-VCH. (C) Schematic representation of the preparation of P*N*IPAAm–ZnPP^(6/6)^HTHP^V44C^, P*N*IPAAmZnPP^(1/6)^HTHP^V44C^, and ZnPP^(*n*/6)^ light-harvesting micelles. Adapted with permission from ref. 107. Copyright 2020, American Chemical Society.

Thermally responsive luminescent materials have great potential for temperature sensing, smart display, and anti-counterfeiting because of their high sensitivity to temperature changes.^3,93,94,103–105^ In some ALHSs, the molecular order of light-harvesting donors shows a significant effect on the light-harvesting efficiency, which can be easily modulated by temperature. Uvdal and coworkers reported an efficient nano-ALHS based on the self-assembly of nanoscale coordination polymers (NCPs), which showed temperature responsiveness for guest capturing and releasing to achieve emission enhancement (Fig. 8A).^106^ The NCPs were prepared by the co-assembly between a linear π-conjugated dicarboxylate (L1) with lanthanide metal ions Gd(iii), Eu(iii), and Yb(iii) in DMF. The guest molecule *trans*-4-styryl-1-methylpyridiniumiodide (D1) or methylene blue (D2) was encapsulated into NCPs. It was found that the D1-loaded NCPs exhibited an efficient light-harvesting process with energy transfer from NCPs to D1. The light-harvesting properties of D1-loaded NCPs were investigated at both 20 °C and 140 °C. The emission color changed from bright blue-violet of L1 to orange after the addition of D1 and Gd(OAc)_3,_ which showed an efficient light-harvesting effect, while the orange emission intensity further increased upon raising the temperature to 140 °C.

Li, Yang and Chen *et al.* have reported a thermochromic ALHS based on anisotropic fluids. In this ALHS, the energy donor is a saddle-shaped discotic liquid crystal compound LC6 bearing a rigid COTh fluorophore and multiple dodecyl chains linked to the periphery, which exhibits excellent temperature-dependent fluorescence chromism (Fig. 8B).^48^ NiR was selected as the energy acceptor that was loaded into this thermochromic liquid crystal platform, and then the light-harvesting efficiency could be tuned at different temperatures by regulating the molecular order of the compound LC6. Meanwhile, not only temperature but also the molar ratio of the donor/acceptor could lead to the variation of the emitting color of such an ALHS. A high AE of up to 39.1 was achieved when the ratio between LC and NiR reached 100 : 1. Furthermore, during the cooling process of the 10 : 1 LC6-NiR light-harvesting system from 80 °C to −30 °C, the fluorescence spectra underwent a red shift and the fluorescence color changed from yellow to red.

Besides, Hayashi, Uchihashi, and Oohora *et al.* developed another method to construct thermo-responsive nano-ALHSs (Fig. 8C).^107^ A mutant of a thermostable hemoprotein, hexameric tyrosine-coordinated heme protein (HTHP) was employed to generate a protein assembly under heating by reacting with maleimide-tethering thermoresponsive poly(*N*-isopropylacrylamide), P*N*IPAAm. It was found that the P*N*IPAAm-modified HTHP (P*N*IPAAm–HTHP) can self-assemble into a 43 nm spherical structure at 60 °C while disassembling at 20 °C. The temperature controlled self-assembly can occur reversibly at least 5 times. A photosensitizer Zn protoporphyrin IX, ZnPP, was loaded into a solution of apoHTHP^V44C^ (a type of HTHP), and further modified with maleimide-terminated P*N*IPAAm (P*N*IPAAm–MI) to obtain P*N*IPAAm–ZnPP^(*n*/6)^HTHP^V44C^. The P*N*IPAAm–ZnPP^(*n*/6)^HTHP^V44C^ was also found to show thermoresponsive assembling behavior, which could provide a micellar assembly (ZnPP^(*n*/6)^ micelle) after heating. To evaluate the energy migration, the fluorescence quenching, fluorescence lifetime and fluorescence anisotropy decay were characterized. It was revealed that energy migration within the ZnPP-substituted micelle occurred within several tens of picoseconds.

### 3.3 pH-Responsive supramolecular nano-ALHSs

**Fig. 9:**
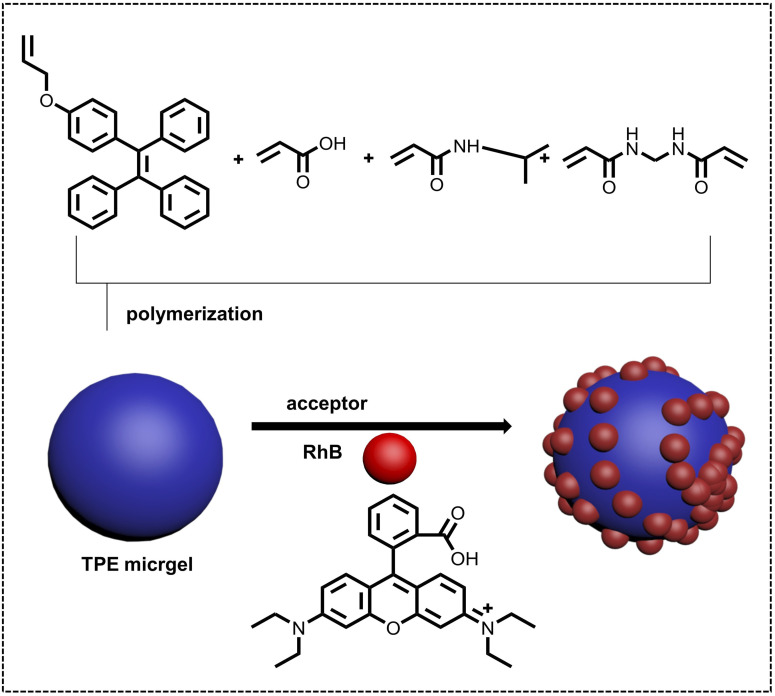
Schematic illustration of the construction of a temperature and pH responsive nano-ALHS based on synthetic AIE-active microgels with TPE AIEgens. Reproduced with permission from ref. 108. Copyright 2021, Wiley-VCH.

pH sensors are often employed to detect pH variation, and are widely used for evaluating water quality, sensing blood, *etc.* Due to the strong emission and good dispersity at a trace amount of luminescent acceptor of ALHSs in water, ALHSs have greater potential applications with higher sensitivities than traditional pH sensors. Therefore, not only in pH sensors but also in pH controlled photocatalysis, pH-responsive ALHSs have attracted much attention. Liu, He and coworkers reported a highly emissive microgel showing both pH and temperature responsiveness (Fig. 9).^108^ The microgel was synthesized by polymerizing TPE based comonomers, acrylic acid, *N*-isopropylacrylamide (*N*IPAM), and permanent crosslinker ethylenebisacrylamide (BIS), which acted as the donor with energy transfer to the acceptor RhB. The pH value showed an obvious effect on the energy transfer efficiency for this light-harvesting system, increasing from 21.6% (pH = 2.0) to 52.3% (pH = 8.0) accompanying the diverse luminescent colors at a molar ratio of TPE/RhB of 5 : 1.

### 3.4 Nano-ALHSs controlled by donor/acceptor ratios

**Fig. 10:**
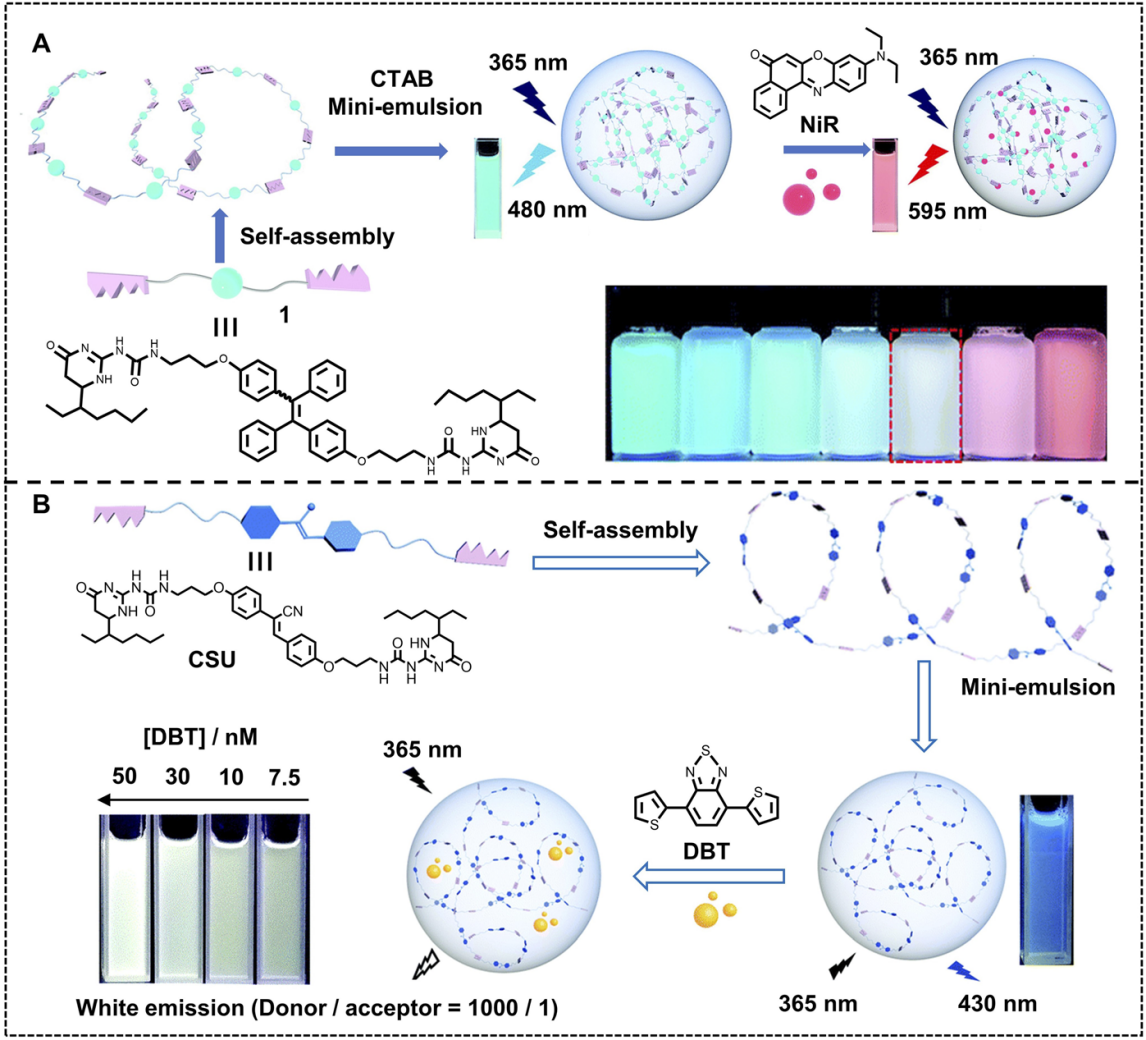
(A) Illustration of the construction of AIE-active microgels based on compound 1, further application of NiR to form nano-ALHS, and photographs of nanoparticles in water with different concentrations of NiR. Reproduced with permission from ref. 109. Copyright 2020, The Royal Society of Chemistry. (B) Schematic illustration of the construction of a white-light emission light-harvesting system. Reproduced with permission from ref. 110. Copyright 2022, The Royal Society of Chemistry.

Undoubtedly, the molar ratio of the donor and acceptor is a key factor for ALHSs to determine light-harvesting efficiency as well as the emergent luminescence because it is an entry point for the construction of each ALHS. The *Φ*_ET_ can be regulated by the molar ratio of the donor and acceptor, and the emission intensity of the acceptor will also change accordingly. Therefore, upon well matching between the donor and acceptor, some ALHSs can facilely change their luminescent colors by varying the molar ratio of the donor and acceptor. Wang, Xiao and coworkers constructed a nano-ALHS through the co-assembly of a typical cationic amphiphile, CTAB, and a polymerized UPy-functionalized TPE derivative (1) as a donor and NiR as an acceptor (Fig. 10A).^109^ The tunable emission behavior of the light-harvesting 1-NiR nanoparticles has been achieved by changing the added amount of NiR. Initially, the nanoparticles of 1 in water showed light blue emission color without NiR. When the addition of NiR was increased as the molar ratio of the donor and acceptor decreased from 1500 : 1 to 100 : 1, the emission color changed from light blue to bright red, meanwhile a strong white emission emerged when the molar ratio reached 250 : 1. Based on this research, they further developed another nano-ALHS to achieve controllable luminescence (Fig. 10B).^110^ In this system, a bifunctional monomer (CSU) with AIE properties was synthesized by linking cyanostilbene (CS) with ureidopyrimidinone (UPy) units, which can self-assemble into a supramolecular polymer. The CSU acts as the donor and DBT as the energy acceptor. By changing the addition amount of DBT, the luminescence color showed a variation from blue to yellow. A white light emission was also realized when the molar ratio between donor and acceptor reached 1000 : 1.

**Fig. 11:**
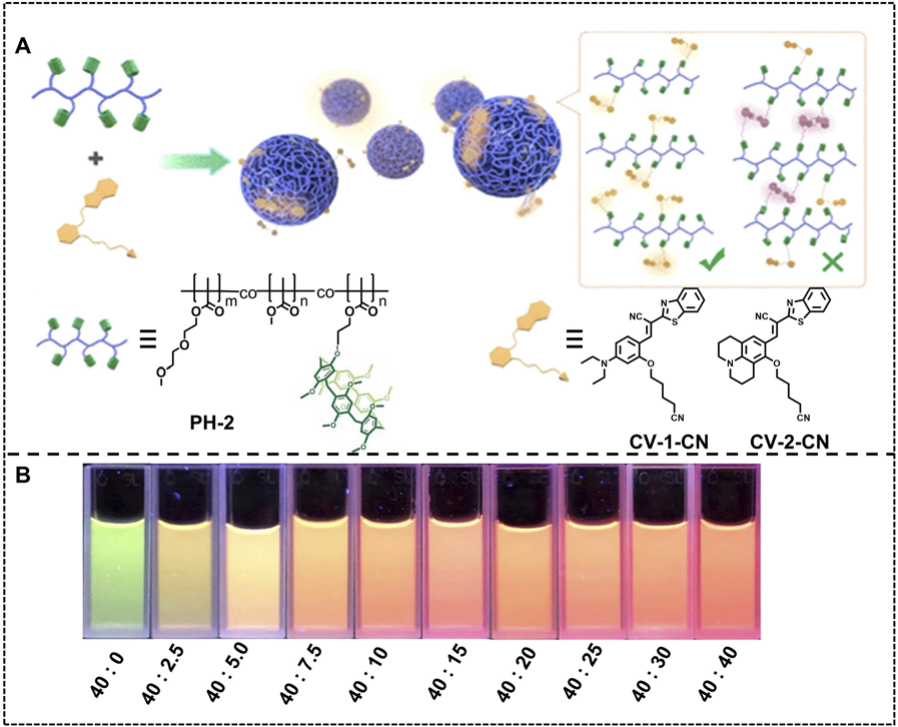
(A) Schematic illustration of SPNs formed by CV derivatives and pillar[5]arene-based polymer hosts: formation of supramolecular nanoparticles by synergetic combination of host–guest interactions and hydrophobic interactions and chemical structures of polymer hosts and CV derivatives. (B) Photographs of CV-2-CN&NiR⊂PH-2 SPNs with various concentrations of CV-2-CN and NiR under excitation of 365 nm light. Reproduced with permission from ref. 111. Copyright 2021, American Chemical Society.

More broad luminescence changing ranges are achieved when we employ luminophores with more luminescent colors as the donors and acceptors. Except for the luminescence color changing from blue to red or yellow, the green to orange color is also obtained by regulating the donor/acceptor ratio of a green emitting donor and an orange emitting acceptor. Yang, Tang and coworkers fabricated a nano-ALHS by employing luminescent supramolecular polymer nanoparticles with cyanovinylene (CV)-based chromophores (CV-1-CN and CV-2-CN) as donors and NiR as an acceptor, whereby efficient FRET processes occur and broaden the initial emission spectra (Fig. 11).^111^ CV-2-CN was included in the cavity of pillar[5]arene from a pillararene-based polymer (PH-2) through host–guest complexation. When the molar ratio between CV-2-CN and NiR ranged from 40 : 0 to 40 : 40, the emission color of the light-harvesting system CV-2-CN&NiR⊂PH-2 supramolecular polymer nanoparticles changed from green to orange.

**Fig. 12:**
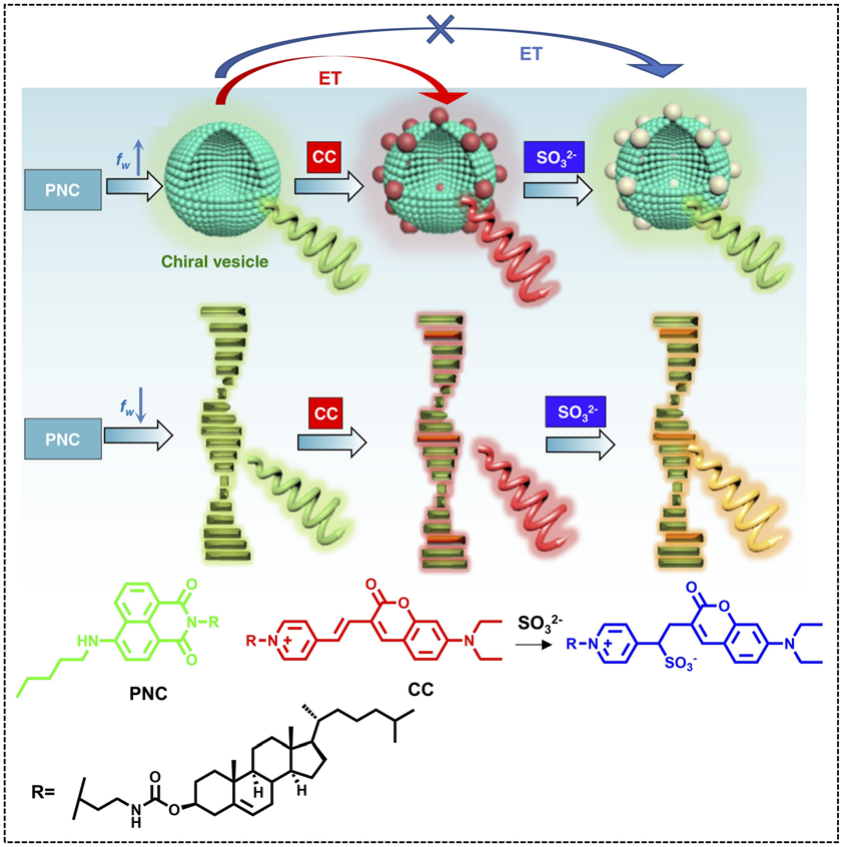
Molecular structures of PNC and CC as well as the anion-responsive energy transfer effect in vesicle and helical fiber structures mediated by the water fraction (*f*_w_) (energy transfer (ET), pentylamine-substituted cholesteryl naphthalimide (PNC), and cholesteryl coumarin (CC)). Blue, red, and gray particles represent PNC, CC and CC* respectively in the vesicle phase. Green, red, and orange rods stand for PNC, CC and CC* respectively in chiral fiber self-assembly. Spiral arrows represent CPL with the corresponding colors. Reproduced under terms of the CC-BY license from ref. 112. Copyright 2021, published by Springer Nature.

The light-harvesting effect of ALHSs can also be controlled by ionic strength. Xing and coworkers presented a co-assembled multi-component system with switchable fluorescence as well as CPL that also showed response to SO_2_ derivatives (Fig. 12).^112^ Pentylamine-substituted cholesteryl naphthalimide (PNC) and a cholesteryl coumarin (CC) derivative were used to co-assemble into vesicles and nanohelices and acted as the energy transfer donor and acceptor, respectively. With different molar ratios between PNC and CC, the emission color changed from green to yellow, orange, and finally to red; meanwhile, the emission wavelength red shifted from 542 nm to 624 nm. The *Φ*_ET_ reached 44.8% (544 nm) at a donor/acceptor ratio of 100 : 5. The chiroptical properties of this system were also investigated. The results showed that energy transfer between PNC and CC allowed for CPL color evolution from green to red depending on the fraction of CC. And after adding SO_3_^2−^, the CPL exhibited a blue shift to 540 nm, which demonstrated that an anion-responsive CPL-active ALHS was achieved.

## 4. Applications of self-assembled supramolecular nano-ALHSs

### 4.1 Anti-counterfeiting

**Fig. 13:**
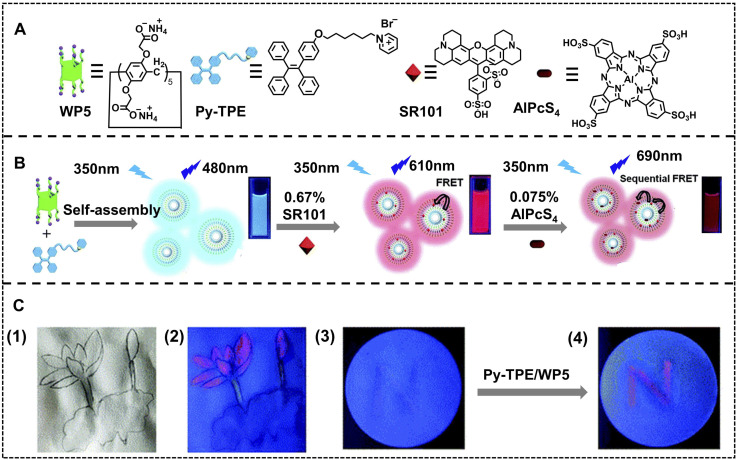
(A) Compounds involved in construction of a light-harvesting system with sequential energy transfer in aqueous solution, including WP5, Py–TPE, SR101 and AlPcS_4_. (B) Schematic illustration for the construction of a nano-ALHS. (C) The fluorescent inks based on a light-harvesting system: (1) photographs under natural light and (2) under UV light of the painting and (3) photograph of writing “N” with SR101 merely and (4) rewriting “N” with Py–TPE/WP5 solution under UV light. Reproduced with permission from ref. 118. Copyright 2020, Royal Society of Chemistry.

Anti-counterfeiting is well-known in modern human society, which avoids financial forgeries and the harm of fake and shoddy products.^113–117^ Benefiting from controllable luminescence, ALHSs can be used as luminescent ink to encrypt information for anti-counterfeiting, namely, information that is not observed under natural light but appears under special light, which is superior to typical fluorescent inks due to the tunable luminescent color and high sensitivity resulting from the trace amount of the acceptor. Liu and coworkers have developed an anti-counterfeiting-oriented highly efficient nano-ALHS based on a supramolecular assembly constructed pillar[5]arene (WP5) with a pyridinium modified TPE derivative (Py–TPE) (Fig. 13).^118^ In this artificial light-harvesting system, Py–TPE/WP5 acted as a donor because of the AIE effect of Py–TPE induced by WP5, which finally resulted in aggregation induced emission enhancement. Then, SR101 was selected as the first acceptor, which could be packaged into the hydrophobic layer of the Py–TPE/WP5 supramolecular co-assembly with a donor/acceptor ratio of 150 : 1 to achieve highly efficient FRET. Finally, the second acceptor, AlPcS_4_, was employed to form a two-step sequential energy transfer accompanied by fluorescence variations from blue to near-infrared (NIR) emission. Moreover, this artificial light-harvesting system with NIR emission was used as fluorescent security ink. The word ‘N’ was written using SR101 on a paper with negligible fluorescence under UV light, while a red fluorescent ‘N’ appeared when Py–TPE/WP5 supramolecular assembly solution was written on the same position of this special paper. Thus, information encryption is realized for use in the anti-counterfeiting field. Among all the secret information, a fingerprint is one of the most important pieces of individual information, which can be used as personal security measures and even for personal identification in criminal cases under some circumstances.^119^

### 4.2 Cell imaging

**Fig. 14:**
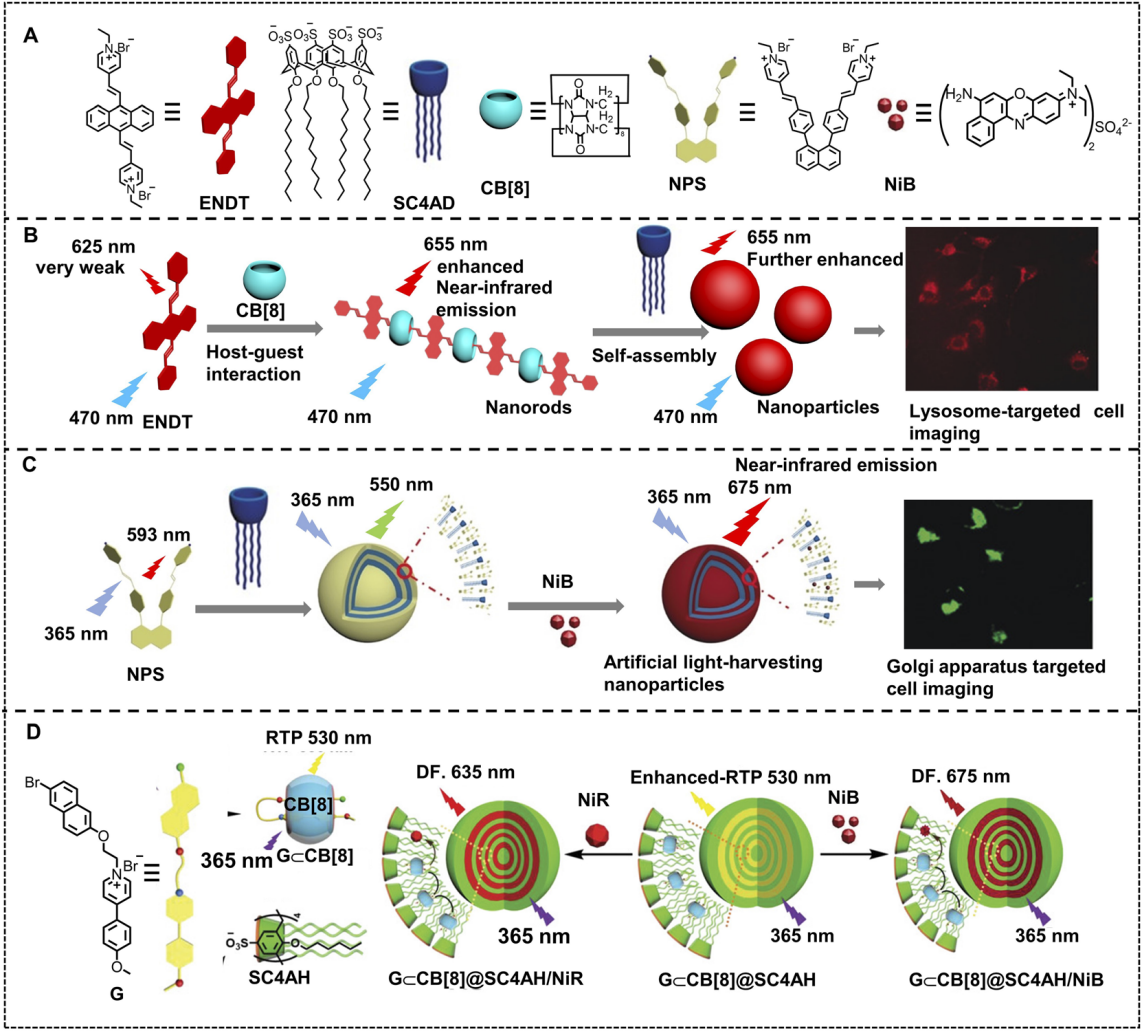
(A) Schematic illustration of building blocks of NIR emissive supramolecular self-assemblies: ENDT, SC4AD, NPS and NiB. (B) Schematic of two-stage enhancement of NIR fluorescent supramolecular assemblies for imaging lysosomes of A549 cells. Reproduced with permission from ref. 122. Copyright 2018, Wiley-VCH. (C) Schematic illustration of the assembly process of the NIR emissive ALHS, NPS–SC4AD–NiB, for Golgi apparatus targeted cell imaging. Reproduced with permission from ref. 123. Copyright 2020, Wiley-VCH. (D) Schematic illustration of the construction of a RTP-capturing system with delayed NIR emission in aqueous solution. Reproduced with permission from ref. 127. Copyright 2022, Wiley-VCH.

Fluorescent nanomaterials are developed as one of the research frontiers of imaging applications, which can provide entrapment space for drug loading and delivery to realize integration of diagnosis and treatment. To enhance luminescence instead of fluorescence quenching in an aggregation state from nanosystems is a key issue for cell imaging oriented fluorescent nanomaterials.^74,76,120,121^ Liu and coworkers developed a two-stage mediated near-infrared emissive supramolecular assembly for lysosome-targeted cell imaging. 4,4′-Anthracene-9,10-diylbis(ethene-2,1-diyl)bis(1-ethylpyridin-1-ium)bromide (ENDT) with weak fluorescence emission at 625 nm, was selected as a guest molecule for cucurbit[8]uril (CB[8]) to form a supramolecular assembly (Fig. 14B).^122^ The fluorescence emission of this supramolecular assembly was enhanced and red shifted to 655 nm because of J-aggregation of ENDT induced by CB[8]. Then, the ENDT&CB[8] assemblies were used to assemble with lower-rim dodecyl-modified sulfonatocalix[4]arene (SC4AD), resulting in a second-stage enhancement of NIR emission. Such co-assemblies were demonstrated to image the lysosome by highly consistent co-staining with Lysotracker blue in human lung adenocarcinoma cells (A549 cells). Because of their many excellent properties such as good light stability, strong and multicolor luminescence, and aggregation without quenching, supramolecular ALHSs possess huge potential for cell-imaging, which has inspired a hot research direction. Li, Yang and coworkers have reported a highly efficient near-infrared emissive supramolecular nano-ALHS for imaging in the Golgi apparatus (Fig. 14C).^123^ A naphthyl-1,8-diphenyl pyridinium derivative (NPS) has been synthesized and used as a fluorescent donor with a weak fluorescence emission at 593 nm. Then, NPS co-assembled with SC4AD to form nanoparticles, exhibiting a sharp aggregation induced emission enhancement with an obvious blue shift to 550 nm. In order to realize near-infrared emission, NiB was loaded into the NPS–SC4AD supramolecular assembly as an energy acceptor due to the sufficient overlap between the absorption band of NiB and the emission band of NPS–SC4AD nanoparticles, which led to an efficient ALHS with 675 nm emission. Furthermore, it has been found that when the donor/acceptor ratio of the NPS–SC4AD–NiB system is 250 : 1 in PBS buffer, the energy transfer efficiency (*Φ*_ET_) and antenna effect (AE) reach up to 60.8% and 33.1, respectively, which manifests that the NPS–SC4AD–NiB system is a highly efficient ALHS. Finally, NPS–SC4AD–NiB nano-ALHS was used in cell imaging and was found to stain the Golgi apparatus in human prostate cancer cells (PC-3 cells). Furthermore, such NPS–SC4AD supramolecular co-assembly light-harvesting platforms are further demonstrated for efficient energy transfer to NiB and NiR, and these ALHSs show synchronous imaging capability in the Golgi apparatus and lysosome.^124^

Taking advantage of its long lifetime, organic room-temperature-phosphorescence (RTP) can greatly avoid the influence of biological fluorescence and background interference, promoting great progress in bio-imaging.^125^ However, there is a limitation to RTP materials; the weak emission caused by amorphous aggregates needs to be solved urgently.^126^ Thus, designing and constructing an ALHS with RTP is promising for solving this problem. Liu and coworkers developed a highly efficient RTP energy transfer nano-ALHS in water (Fig. 14D).^127^ This light-harvesting system was constructed *via* a free bromonaphthalene-connected methoxyphenyl pyridinium salt (G), CB[8] and an amphiphilic calixarene (SC4AH) by a two-stage assembly strategy, exhibiting remarkably enhanced RTP emission at 530 nm. Moreover, two organic dyes, NiB and NiR, were loaded into the hydrophobic layer of G⊂CB[8]@SC4AH as acceptors, respectively, which endowed the light-harvesting system with delayed near-infrared emission at 635 nm and 675 nm. The *Φ*_ET_ and AE of the two RTP light-harvesting systems were further investigated. The *Φ*_ET_ was calculated to be 64.1% and phosphorescent AE was 352.9 at a donor/acceptor ratio of 150 : 1 for the G⊂CB[8]@SC4AH/NiR ALHS. Meanwhile, the *Φ*_ET_ and AE of G⊂CB[8]@SC4AH/NiB were calculated to be 49.6% and 123.5 at a donor/acceptor ratio of 300 : 1. Thus, compared to G⊂CB[8]@SC4AH/NiR, the excellent phosphorescence-capturing capability of the G⊂CB[8]@SC4AH/NiB system will play an important role in cell imaging, which was found to mark lysosomes with relatively good stability in living cells (A549). Except for nano-ALHSs based on supramolecular nanoparticles, light-harvesting systems based on 2D nanosheets can also be successfully constructed for cell imaging.

### 4.3 Photocatalysis

In natural light-harvesting systems, the processes that realize the transformation of solar energy into chemical energy in a series of steps are collectively called photosynthesis. Therefore, mimicking natural photosynthesis is one of the final aims for the research on ALHSs.^67,128–132^

**Fig. 15:**
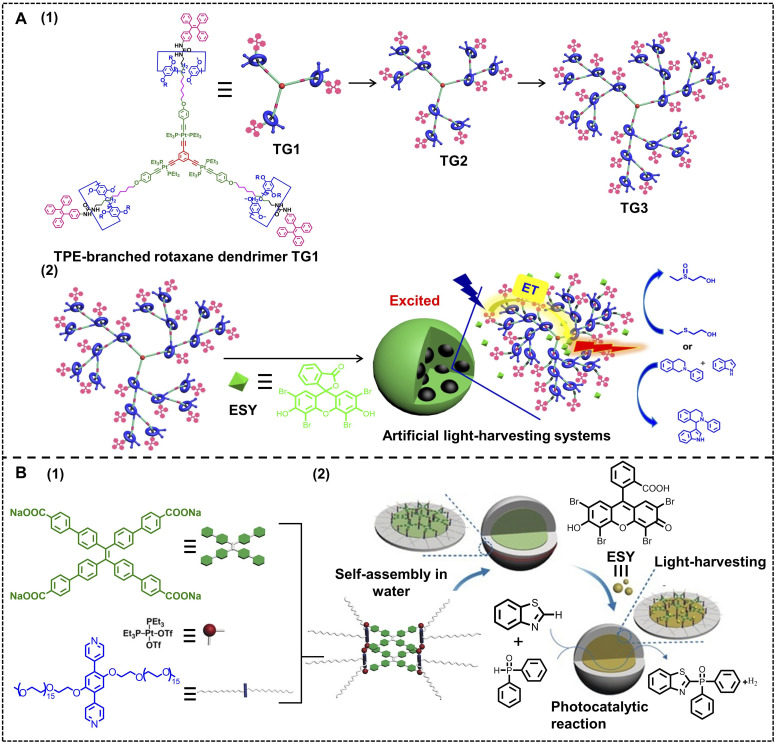
(A) (1) Chemical structure of TG1 and schematic illustration of a controllable divergent strategy for the synthesis of TPE-branched rotaxane dendrimers TG2 and TG3. (2) Schematic illustration of the ALHSs based on rotaxane dendrimers and their catalysis for two types of reactions. Reproduced with permission from ref. 133. Copyright 2021, Wiley-VCH. (B) (1) Fabrication of the metallacage. Tf represents trifluoromethanesulfonyl. (2) Schematic representations of the light-harvesting system and its application in a photocatalytic reaction. Reproduced with permission from ref. 134. Copyright 2019, Wiley-VCH.

Light-harvesting systems can be used as photocatalysts like other traditional catalysts for chemosynthesis. Yang, Wang and coworkers have reported a nano-ALHS for the photooxidation reaction and aerobic cross dehydrogenative coupling reaction (Fig. 15A).^133^ The ALHS is constructed by a TPE-branched rotaxane dendrimer TG*n* (*n* = 1, 2, 3) self-assembling with the fluorescent dye ESY acting as the fluorescence energy acceptor (TG*n*–ESY, *n* = 1, 2, 3). When the molar ratio of TPE/ESY was set as 3 : 1, the *Φ*_ET_ were calculated to be 42.5% for TG1–ESY, 68.2% for TG2–ESY, and 71.6% for TG3–ESY, respectively. As for photooxidation, the TG*n*–ESY ALHSs were supposed to be capable of producing singlet oxygen (O_2_), which guarantees the photocatalyst system for both the photooxidation reaction and aerobic crossdehydrogenative coupling (CDC) reaction. 2-(Ethylsulfanyl)ethanol was selected as a model compound for photooxidation, and the full conversion from sulfide to sulfoxide was achieved after 10.0 h, 8.0 h and 4.0 h of reaction using TG1–ESY, TG2–ESY and TG3–ESY as photocatalysts, respectively. When keeping the same amount of the TPE units, the higher generation TG was proven to have a boosted photocatalytic performance. Furthermore, *N*-phenyl-1,2,3,4-tetrahydroisoquinoline with an indole group was chosen as another model compound for a typical aerobic cross-coupling reaction. The full conversion of the target product was achieved after 11.0 h, 8.0 h, and 4.0 h for TG1–ESY, for TG2–ESY for TG3–ESY as photocatalysts with isolated yields of 95%, 90%, and 89%, respectively, which is consistent with the photooxidation results. Zhang and coworkers have developed a nano-ALHS by employing an emissive poly(ethylene glycol)-decorated tetragonal prismatic platinum(ii) metallacage as the donor and ESY as the energy acceptor in aqueous solution because of the good spectral overlap between the emission band of the metallacage and the absorption band of ESY (Fig. 15B).^134^ A TPE-based metallacage was constructed through the co-assembly of TPE-based sodium benzoate ligands, the dipyridyl ligand with eight poly(ethylene glycol) chains and *cis*-Pt(PEt_3_)_2_(OTf)_2,_ as the donor for the FRET process in aqueous light-harvesting systems. This aqueous ALHS was further used to catalyze the cross-coupling hydrogen evolution reaction for benzothiazole with phosphine oxide. Compared with those obtained when using only ESY, both the conversion of benzothiazole and the yield of benzo[*d*]thiazol-2-yldiphenylphosphine oxide increased dramatically when using the platinum(ii)-cage-based nano-ALHS as a catalyst. After reaction for 12 h, the conversion of benzothiazole reached 88% and the yield of benzo[*d*]thiazol-2-yldiphenylphosphine oxide reached 65%; however, only 40% conversion and 33% yield were obtained with only ESY as the catalyst.

**Fig. 16:**
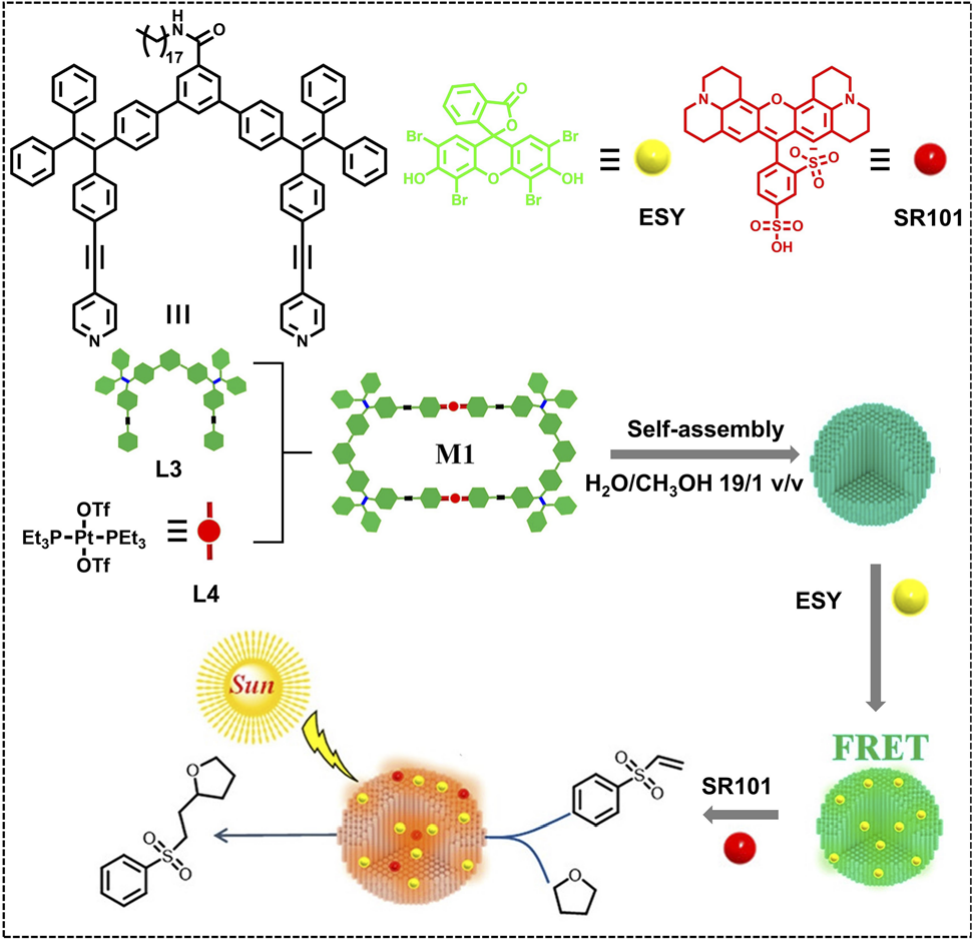
Schematic illustration of the construction of a metallacycle-based ALHS and the processes of light-harvesting and photocatalysis. Reproduced with permission from ref. 135. Copyright 2021, American Chemical Society.

Nano-ALHSs with a two step FRET effect have also been developed for photocatalysis. Yi, Zhang and coworkers have presented a metallacycle-based ALHS with a sequential energy transfer process for the photocatalyst (Fig. 16).^135^ A quadrilateral platinum(ii) metallacycle containing a TPE-based ligand (M1) can self-assemble in H_2_O/MeOH (19/1,v/v) which is used as a donor to build a light-harvesting platform owing to its AIE effect. Two fluorescent dyes, ESY and SR101, were selected as the energy transfer acceptors for the first and second steps, respectively. Then, the two acceptors were loaded into M1 self-assemblies to form the M1–ESY–SR101 ALHS with high efficiency. Subsequently, the alkylation of C–H bonds for phenyl vinyl sulfone and tetrahydrofuran was catalyzed by the M1–ESY–SR101 system in an aqueous medium under irradiation of a Xe lamp at 50 °C. The result of the reaction showed that the conversion of phenyl vinyl sulfone reached 99% and the yield of 2-(2-(phenylsulfonyl)ethyl)tetrahydrofuran is 48% after 1 h of irradiation in H_2_O–THF (19 : 1, v/v) containing 1 mol% of M1–ESY–SR101. In contrast, without SR101, the remaining M1–ESY catalyst only exhibited 21% yield of 2-(2-(phenylsulfonyl)ethyl)tetrahydrofuran, which proved that the two sequential energy transfers can improve catalytic efficiency significantly.

**Fig. 17:**
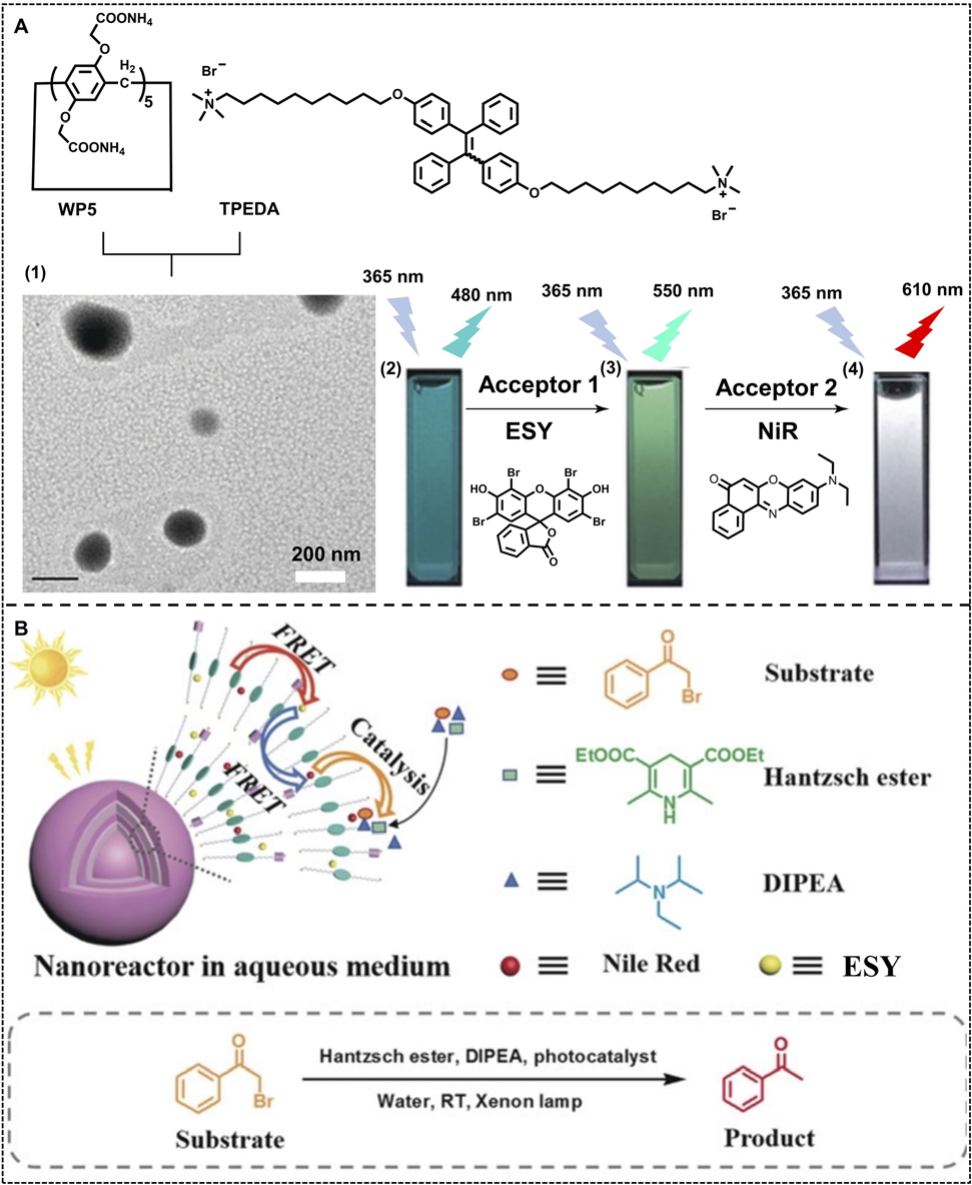
(A) Illustration of the construction of sequential pillar[5]arene-based nano-ALHS, and the chemicals involved are WP5, TPEDA, ESY and NiR. (1) TEM image of nanoparticles formed by WP5 and TPEDA in water, (2) photograph of the aqueous solution with luminescent nanoparticles under 365 nm excitation, (3) variation in the luminescent colour under 365 nm excitation after ESY was applied as the first acceptor, and (4) successful construction of white emission after application of NiR as the second acceptor. (B) Schematic illustration of the dehalogenation reaction using the WP5⊃TPEDA–ESY–NiR assembly as a nanoreactor in an aqueous medium. Reproduced with permission from ref. 136. Copyright 2020, Wiley-VCH.

Wang, Hu and coworkers have also reported a nano-ALHS based on the supramolecular assembly between water-soluble WP5 and a bola-type TPE-functionalized dialkyl ammonium derivative (TPEDA) to achieve a two-step sequential energy-transfer process in aqueous solution (Fig. 17).^136^ The AIE properties of the TPE group makes TPEDA to become a light-harvesting donor, whose emission can be enhanced by co-assembling with WP5 to form supramolecular nanoparticles. The formed emissive nanoparticles can act as a light-harvesting donor platform for energy transfer to an energy acceptor ESY as the first step. Furthermore, NiR was added to realize the second sequential energy-transfer process at a 200 : 1 : 1 molar ratio of TPEDA/ESY/NiR. Furthermore, the WP5⊃TPEDA–ESY–NiR nanoreactor was used to catalyze the dehalogenation reaction of α-bromoacetophenone with Hantzsch ester in aqueous solution. The yield of product acetophenone reached 96% with WP5⊃TPEDA–ESY–NiR after 8 h of irradiation, which increased significantly in comparison to that obtained with other control catalysts. The photobleaching effect and fluorescence quenching of the WP5⊃TPEDA–ESY–NiR nanoreactor were restrained, which led to the high catalytic efficiency of photosynthesis.

## 5. Conclusion and outlook

In this review, we systematically summarize the recent advances in supramolecular nano-ALHSs with a focus on three aspects: construction of supramolecular nano-ALHSs based on different substrates, the modulation strategy for supramolecular nano-ALHSs, and applications of the obtained supramolecular nano-ALHSs. Compared with the covalent bonding between light-harvesting donors and acceptors to achieve ALHSs, supramolecular nano-ALHSs based on non-covalent interactions can simplify the complex synthesis and provide higher light-harvesting efficiency and more flexible options for energy matching between donor and acceptor chromophores. Although non-covalent interactions from supramolecular nano-ALHSs may lead to less stability of the created structures as well as the effect of ALHSs, remarkable efforts and inspiring progress have been made to strengthen supramolecular nano-ALHSs toward meeting the standard of practical applications. Some potential methods are suggested here toward increasing their stability: (1) active reaction precursors can be modified on building blocks, so that *in situ* reactions can be carried out to stabilize the assembled structure after the formation of supramolecular nano-ALHSs; (2) inspired by natural light-harvesting systems that are protected in chloroplasts, we can design a protective enclosure to avoid the degradation of supramolecular nano-ALHSs; (3) because most of the supramolecular nano-ALHSs were constructed in a solution state that can be easily influenced by the environment of the solvents, the design of solvent-free supramolecular nano-ALHSs may serve as a preferable way to construct ALHSs with higher stability.

Natural light-harvesting systems belong to nanosystems, so constructing nano-ALHSs is probably the most desirable and available pathway for mimicking the natural light-harvesting process. In addition, according to the principle of FRET during the light-harvesting process, nano-ALHSs provide a suitable spatial distance (0.1–10 nm) for higher energy transfer efficiency. Moreover, like the natural light-harvesting system that serves as the first step of photosynthesis, one of the most important and rational applications of nano-ALHSs is to realize highly efficient photocatalysis, where not only a large specific surface area but also special bonding and electronic states on the surface of nano-ALHSs can provide for improving the catalytic efficiency and selectivity. Besides photocatalytic applications that are closest to photosynthesis, the captured light energy can be directly utilized for smart display, information encryption and anti-counterfeiting. Although nano-ALHSs do not seem to show obvious advantages in this application area, the molecular order and alignment of supramolecular assemblies in ALHSs possess great potential for advanced information encryption, which depends on the nanostructures of supramolecular ALHSs. Furthermore, from a dimension perspective, only nano-ALHSs could work in living cells. According to this, cell imaging as well as further biological applications offer a promising and interesting research field, not only because of the bright emission obtained from acceptors of nanoscale ALHSs, but also because it endows living beings with the potential to harvest light energy directly from the sun. As is well-known, natural photosynthesis can only occur in higher plants and some photosynthetic bacteria, while most higher animals do not have such an ability. Thus, we can imagine, if animals have the ability to obtain energy through ALHSs in their cells, the efficiency of solar energy will be greatly improved. Overall, in consideration of all the potential applications that are discussed, we expect that supramolecular nano-ALHSs will be very promising to be further developed into fields such as artificial photosynthesis, advanced encryption, integration of diagnosis and treatment, and beyond.

## Conflicts of interest

The authors declare no conflict of interest.

## Supplementary Material
